# The Use of Evaluation Panels During the Development of a Digital Intervention for Veterans Based on Cognitive Behavioral Therapy for Insomnia: Qualitative Evaluation Study

**DOI:** 10.2196/40104

**Published:** 2023-03-06

**Authors:** Arthur Thomas Ryan, Lisa Anne Brenner, Christi S Ulmer, Margaret-Anne Mackintosh, Carolyn J Greene

**Affiliations:** 1 Rocky Mountain Mental Illness Research, Education and Clinical Center for Suicide Prevention Department of Veterans Affairs Aurora, CO United States; 2 Department of Psychiatry University of Colorado Anschutz Medical Campus Aurora, CO United States; 3 Departments of Physical Medicine and Rehabilitation, Psychiatry, and Neurology University of Colorado Anschutz Medical Campus Aurora, CO United States; 4 Center of Innovation to Accelerate Discovery and Practice Transformation Durham Veterans Affairs Health Care System Durham, NC United States; 5 Department of Psychiatry and Behavioral Sciences Duke University School of Medicine Durham, NC United States; 6 National Center for Posttraumatic Stress Disorder Dissemination and Training Division Veterans Affairs Palo Alto Health Care System Palo Alto, CA United States; 7 University of Arkansas for Medical Sciences Translational Research Institute University of Arkansas for Medical Sciences Little Rock, AR United States

**Keywords:** cognitive behavioral therapy for insomnia, CBT-I, insomnia, digital mental health intervention, digital MH intervention, internet-delivered, veterans, Path to Better Sleep

## Abstract

**Background:**

Individuals enrolling in the Veterans Health Administration frequently report symptoms consistent with insomnia disorder. Cognitive behavioral therapy for insomnia (CBT-I) is a gold standard treatment for insomnia disorder. While the Veterans Health Administration has successfully implemented a large dissemination effort to train providers in CBT-I, the limited number of trained CBT-I providers continues to restrict the number of individuals who can receive CBT-I. Digital mental health intervention adaptations of CBT-I have been found to have similar efficacy as traditional CBT-I. To help address the unmet need for insomnia disorder treatment, the VA commissioned the creation of a freely available, internet-delivered digital mental health intervention adaptation of CBT-I known as Path to Better Sleep (PTBS).

**Objective:**

We aimed to describe the use of evaluation panels composed of veterans and spouses of veterans during the development of PTBS. Specifically, we report on the methods used to conduct the panels, the feedback they provided on elements of the course relevant to user engagement, and how their feedback influenced the design and content of PTBS.

**Methods:**

A communications firm was contracted to recruit 3 veteran (n=27) and 2 spouse of veteran (n=18) panels and convene them for three 1-hour meetings. Members of the VA team identified key questions for the panels, and the communications firm prepared facilitator guides to elicit feedback on these key questions. The guides provided a script for facilitators to follow while convening the panels. The panels were telephonically conducted, with visual content displayed via remote presentation software. The communications firm prepared reports summarizing the panelists’ feedback during each panel meeting. The qualitative feedback described in these reports served as the raw material for this study.

**Results:**

The panel members provided markedly consistent feedback on several elements of PTBS, including recommendations to emphasize the efficacy of CBT-I techniques; clarify and simplify written content as much as possible; and ensure that content is consistent with the lived experiences of veterans. Their feedback was congruent with previous studies on the factors influencing user engagement with digital mental health interventions. Panelist feedback influenced multiple course design decisions, including reducing the effort required to use the course’s sleep diary function, making written content more concise, and selecting veteran testimonial videos that emphasized the benefits of treating chronic insomnia symptoms.

**Conclusions:**

The veteran and spouse evaluation panels provided useful feedback during the design of PTBS. This feedback was used to make concrete revisions and design decisions consistent with existing research on improving user engagement with digital mental health interventions. We believe that many of the key feedback messages provided by these evaluation panels could prove useful to other digital mental health intervention designers.

## Introduction

### Background

Approximately 10% of the general population reports insomnia with daytime functional impairment [1]. However, clinically significant insomnia is even more common among veterans, with >50% of veterans enrolling for care in the Veterans Health Administration (VHA) reporting symptoms consistent with a diagnosis of insomnia disorder [2]. Although insomnia itself can cause considerable distress, untreated insomnia is also associated with an increased risk for a variety of other negative outcomes, including suicidal ideation [3], depressive episodes [4], other psychiatric and substance use disorders [5], cardiovascular disease, type 2 diabetes, and all-cause mortality [6], as well as decreased cognitive functioning [7], lower occupational functioning [8], and reduced quality of social relationships [9].

Unfortunately, the most commonly prescribed treatments for insomnia (ie, sleep hygiene education and hypnotic medications) are, at best, relatively ineffective and, at worst, potentially harmful (eg, the use of benzodiazepines to treat insomnia in older adults) [10-12]. In contrast, multiple systematic reviews and meta-analyses have found that cognitive behavioral therapy for insomnia (CBT-I) is a highly effective treatment for insomnia [13], including individuals with comorbid depression, anxiety, or trauma-related disorders [14-17]. Consistent with this research, major sleep medicine societies (eg, the American Academy of Sleep Medicine and the European Sleep Research Society) have designated CBT-I as the gold standard treatment for insomnia [11,18]. Within the Department of Veterans Affairs and Department of Defense Guidelines for The Management of Chronic Insomnia Disorder and Obstructive Sleep Apnea, CBT-I is strongly recommended for the treatment of chronic insomnia disorder [19].

The VHA has sought to disseminate CBT-I throughout its many facilities via the development of standardized educational materials and deployment of trainers to train providers in CBT-I [20]. This dissemination effort has been successful, with >1000 providers trained in CBT-I. Significant improvements in insomnia symptoms, depression symptoms, and quality of life have been noted among veterans who have received this intervention [21]. Despite these efforts, the availability of trained CBT-I providers continues to limit the number of individuals who can receive CBT-I treatment, particularly outside of the VHA, where many veterans receive their health care [22,23]. Other factors can also limit veterans’ ability to receive CBT-I treatment, including the need to travel for repeated therapy appointments, which are primarily available during regular business hours [24].

### Developing a VA Digital Mental Health Intervention Adaptation of CBT-I

Meta-analyses have found that digital mental health (MH) interventions that adapt traditional CBT treatment protocols are effective in treating a wide variety of MH conditions [25]. Digital MH interventions have the potential to address some of the shortcomings of face-to-face psychotherapy, including cost-efficiency and providing easier access for individuals who live in remote locations [26]. With regard to insomnia disorder, several digital CBT-I (dCBT-I) interventions have been developed. Meta-analyses of dCBT-I trials have found that dCBT-I interventions have similar treatment efficacy to traditionally delivered CBT-I [27]. dCBT-I has also been found to be effective in trials with active-duty military personnel [28].

In 2018, the Veterans Affairs (VA) Office of Mental Health and Suicide Prevention commissioned the creation of a dCBT-I intervention for its existing “Veteran Training” platform, a VA website that hosts other digital MH interventions and is available to both veterans and members of the public [29]. A team of VA clinicians, researchers, and administrators was organized to create this new dCBT-I intervention, which was named Path to Better Sleep (PTBS) [30]. To maintain consistency with existing VA CBT-I protocols and to ensure a veteran-centric approach, PTBS content was adapted from an existing VA CBT-I self-management workbook developed by Ulmer et al [31]. A disabled veteran–owned small business, which had previously developed digital MH interventions for the Veteran Training platform, was selected to design and develop PTBS under the direction of the VA team.

### Previous Research on Gathering User and Other Stakeholder Feedback to Inform Digital MH Intervention Development

Soliciting user and other stakeholder feedback is a key element of many digital MH intervention design frameworks, including person-based [32], holistic [33], user-centered [34], and iterative [35]. These frameworks suggest that using user and other stakeholder feedback helps improve the usability of digital interventions, user engagement (ie, uptake and sustained interaction with interventions [36]) and similar metrics (eg, adherence). Increasing user adherence (and similar metrics) to digital MH interventions is important because it is associated with increased therapeutic efficacy [37].

Across published studies that include stakeholder feedback on digital MH interventions, a wide variety of data collection methodologies have been used, including questionnaires, “think-alouds” (ie, having users narrate their thoughts aloud while using a digital MH intervention), interviews, and focus groups [35]. This range of data collection methodologies likely reflects that different methodologies can provide complementary information. For example, questionnaires can provide quantitative data on the overall perceived usability, but qualitative methods allow for eliciting user reactions to specific elements of the intervention [35].

### This Paper

In line with the recommendations made by several digital MH intervention design frameworks, during PTBS’s development, the VA development team used an iterative process of soliciting input from veterans and their family members so that PTBS design and content (ie, the text, illustrations, videos, and interactive exercises used to teach the user how to use the techniques taught in traditional CBT-I) could be tailored to veterans with the goal of improving user engagement with the final intervention. To this end, the VA engaged a communications firm (specifically, a second disabled veteran–owned small business) to convene evaluation panels of veterans and spouses of veterans to provide feedback on drafts of PTBS content. The questions and prompts used during the evaluation panels were intended to solicit feedback on aspects of PTBS so that changes could be made to facilitate user engagement with the completed intervention.

This paper describes the methods used to conduct the PTBS evaluation panels, the feedback gathered from those panels, how that feedback informed the development of PTBS, and the broader lessons that might be drawn about developing digital MH interventions.

## Methods

### Recruitment and Procedure

The panelists were recruited from the contracted communication firm’s networks of individuals and organizations within military and veteran communities. The panelists represented the US Air Force, US Army, US Marine Corps, and US Navy, and were diverse in terms of age, gender, race, rank, and geography. The veterans were not required to have received services from the VA to participate in the panels. Neither veterans’ identities nor any personally identifiable information was disclosed to the members of the VA team. The detailed inclusion criteria are summarized in Textbox 1. Three panels of 9 veterans (n=27) and 2 panels of 9 spouses of veterans (hereafter “spouses”; n=18) were convened monthly for 3 consecutive months (ie, March, April, and May 2017). The marital partners of spouse panelists were not eligible to serve as veteran panelists.

Several choices needed to be made when deciding on the specific procedures for gathering user and stakeholder feedback. The VA team chose to use the evaluation panel methodology because it allowed for rapidly gathering qualitative feedback from a number of veterans and their spouses on many different aspects of PTBS content (including interface design, writing style, consistency with stakeholders’ values, etc). The VA team chose to have each panel meet once a month for 3 months so that there would be time to process feedback from the previous month’s meetings before the next month’s meetings would be held. In this way, panelists could be asked about proposed solutions to the issues raised during the previous month’s meeting. In addition, as the development of PTBS continued during the 3-month period when panel meetings were conducted, the spacing between meetings gave the VA team and contractors time to produce additional content for the course that could be reviewed by the evaluation panels. The 1-hour duration of the panels was chosen because this is a standard recommended duration for evaluation panel meetings to ensure sufficient time to gather feedback without excessively fatiguing the panelists [38].

Spouses were recruited to be panelists as previous research (and the VA team members’ clinical experience) has found that spouses and other family members can have an important influence on whether veterans use MH care [39]. Gathering feedback from spouses was also consistent with the recommendations of digital MH intervention design frameworks that encourage soliciting feedback from stakeholders who play a role in the usage of the intervention by users [32]. Panelists were grouped into veteran-only and spouse-only panels to enable spouses speaking as openly as possible about their positive and negative experiences with facilitating health care use and behavior change in their veteran partners (which they might feel less free to discuss if veterans were present on the panel). Having separate veteran and spouse panels also allowed the VA team and meeting facilitators to tailor the questions and discussion prompts specifically to veteran-only and spouse-only groups.

**Panelist screening and selection criteria.**

**Panelist screening**

- In the case of spousal panelists, the selection criteria were applied to the spouse’s marital partner unless otherwise indicated.
- A veteran with active-duty experience in the US Armed Forces with preference for combat experience
- Age 18 to 70 years
- At least 1 to 2 officers were to be included across all panels.
- A mix of veterans who served after September 11, 2001, and those who served prior
- Inclusion of both urban and rural veterans
- At least 1 female veteran per panel and 1 to 2 male spouses across both spouse panels
- Representation from the US Army, US Navy, US Air Force, and US Marine Corps

**Selection criteria**

- All panelists had to answer “yes” to the question: “Have you ever utilized online resources on your computer or smartphone for educational, self-help, or any other kind of self-guided opportunity for learning, growth or change?”
- All panelists had to report that they owned and used a smartphone.
- All spouse panelists had to have been in a committed relationship with a US Armed Services veteran for >1 year.
- All veterans who were discharged must have done so honorably or on a general discharge.
- One to two active duty or reservists were allowed per group.
- All panelists had to agree with at least 1 of the following 2 statements:
  “I am familiar with some of the challenges facing veterans when they reintegrate into civilian life.”
  “I or someone close to me has been personally affected by insomnia, irritability, stress, addiction, or another mental health or personal issue.”

### Ethical Considerations

A protocol for the preparation of this manuscript was submitted to the Colorado Multiple Institutional Review Board, which serves as the institutional review board for the VA Eastern Colorado Health Care System and other affiliated local institutions. The Colorado Multiple Institutional Review Board certified that the preparation of this manuscript was not considered human subjects research and that institutional review board approval was not required for preparing the present manuscript because (1) the panels served the function of program evaluation for a VHA product development project, not the function of research, and (2) the identities of the veteran and spouse panelists were kept private by the communications firm and were not shared with the VA. All panelists provided their permission, in writing, to be interviewed as part of their participation in the evaluation panels. The firm offered small panelist stipends to participate.

### Procedure

The communications firm that recruited the panelists also organized panel meetings. Two firm employees facilitated the meetings and recorded panelists’ feedback. Panels were conducted using an Adobe Connect virtual meeting room (for the display of visual material) and a telephone conference line (audio). The panelists did not have webcams and could not see each other. Panels were 60 minutes long. To encourage frank and honest discussions, the panelists were asked to keep the views shared by other panelists confidential and were addressed simply by their first name and city of residence during the panels (no members of the same panel resided in the same city).

Before each month’s round of panels, members of the VA team created a description of the content they wanted the panelists to provide feedback on. The communications firm then prepared a facilitator guide for use during the panels. The VA team reviewed the guide, requested revisions if needed, and approved the final draft of the guide. The guides provided facilitators with a detailed script on what to say and instructions on how to facilitate panelist discussion. The guides explicitly organized the prompts into topic areas (eg, the barriers panelists encountered when trying to use web-based resources in the past). The topics assessed in each month’s panel meetings are presented in Textbox 2. In the first month, veteran and spouse panels were asked different questions about the same topic (eg, veterans were asked about their previous experiences making considerable behavioral changes, while spouses were asked about their previous experiences helping their veteran partners make considerable behavioral changes). In the second and third months, nearly identical prompts were used for the veteran and spouse panels. The facilitator guides for each month’s panels are presented in Multimedia Appendices 1-6.

Members of the VA team listened silently to the panel meetings. The facilitators took detailed contemporaneous notes. After the panel, the facilitators verified and expanded upon the notes using audio recordings of the proceedings. The facilitators then aggregated, categorized, and coded the panelists’ feedback according to the structure laid out in the facilitator guide (eg, feedback to prompt 1 or feedback to prompt 2). When the data from a month’s panels were processed in this manner, the facilitators prepared a draft monthly summary report that summarized the feedback from those panels. This draft monthly summary report was then forwarded to the VA team, which reviewed the draft and requested further information or clarification as needed. After any requested revisions were made, a final monthly report was submitted. At the end of the 3 months of panels, the facilitators also prepared a final summary report that summarized feedback from across all 3 months of panels; the 3 monthly reports and the final summary report were 80 pages in total. The descriptions of the panelists’ feedback recorded in these reports served as the raw material from which this manuscript was prepared.

**Key topics during each round of panels.**

**Round 1: veteran panels**

1. Perceived motivators, facilitators, and barriers to uptake and persistence with self-help resources on the web.
2. Decision-making and follow-through when making significant life changes.

**Round 1: spouse panels**

1. Their ability and willingness to influence their veterans to seek outside help or resources (particularly web-based resources).
2. The process of finding resources and determining which ones they would recommend to their veterans.
3. Their perceived role in helping their veterans make significant life changes.

**Round 2: veteran and spouse panels**

1. The layout, text, and functionality of drafts of Path to Better Sleep (PTBS) content, especially the sleep diary, sleep prescription calculator, and relapse prevention plan.
2. How panelists would describe the sleep diary and other PTBS components to others.

**Round 3: veteran and spouse panels**

1. The layout, text, and functionality of drafts of PTBS content, especially the landing page, course guide map, initial learning module, and fact sheet.

### Visual Materials Shown During Evaluation Panels

**Figure 1:**
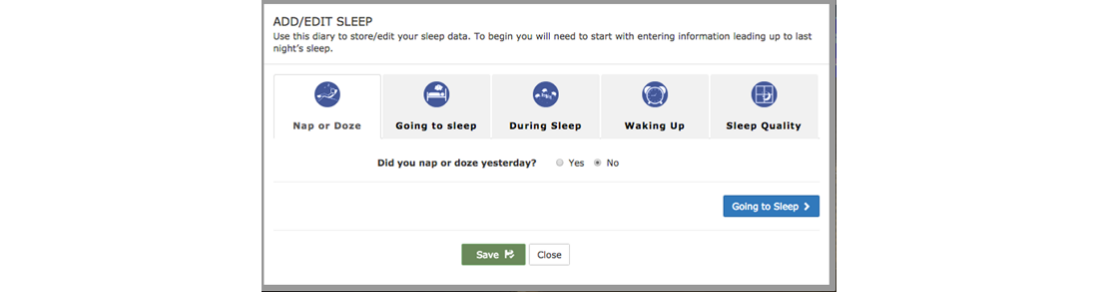
Example of Path to Better Sleep draft material and corresponding feedback prompts.

A contracted private software development firm programmed PTBS. During the panels, content from the underdevelopment PTBS intervention was shown to the panelists to elicit feedback on content and design. Screenshots of the PTBS prototype were shown in some cases. In others, a prototype web page was manipulated by the facilitator to demonstrate its functionality (eg, entering sleep data into the sleep diary). An example of a screenshot shown to the panelists and the feedback questions associated with it is shown in Figure 1. Additional visual content shared with the panelists can be found in the facilitator guides in Multimedia Appendices 1-6. During the evaluation panels, multiple-choice polling questions were occasionally administered to panelists.

### Feedback Prompts

The following are feedback prompts given to panelists along with the screenshot from PTBS shown in Figure 1.

- In looking at the setup of the Sleep Diary page, what do you think of the flow of this page?
- What are your thoughts on the names and titles of each section (Nap or Doze, Going to Sleep, etc)?
- What would you like to see added or clarified?
- What would you do next?

## Results

### Panelists

Three 9-member veteran panels (n=27) and two 9-member spouse panels (n=18) were successfully recruited. Consistent with their previous experience recruiting veteran evaluation panels, the contracted firm did not report problems in recruiting the desired number of panelists. Table 1 reports the demographics of the panel members. The meeting facilitators reported that nearly all panelists arrived on time for panel meetings and participated actively. The percentages of panelists returning for the second- and third-month panels were as follows: second-month return rate for veterans was 89% (24/27) and spouses was 72% (13/18); third-month return rates for veterans was 89% (24/27) and spouses was 83% (15/18).

**Table 1:** Panel demographics.

		Veterans, n	Spouses, n	Veteran partners of spouse panelists^a^, n
Participants		27	18	18
**Self-identified gender**				
	Male	12	2	N/A^b^
	Female	12	13	N/A^b^
	Unavailable^c^	3	3	N/A^b^
**Age range (years)**				
	18-25	0	1	0
	26-30	6	4	4
	31-35	6	3	4
	36-40	3	2	0
	41-45	4	2	4
	46-50	4	2	1
	51-55	2	3	3
	56-60	2	1	1
	>60	0	0	1
Age (years), median		38	36	41.5
**Rank**				
	Enlisted	20	N/A^d^	14
	Officer	7	N/A	4
**Service branch**				
	US Air Force	4	N/A	2
	US Army	9	N/A	12
	US Marine Corps	7	N/A	3
	US Navy	7	N/A	1
**Household income range (US $)**				
	<25,000	2	2	—^e^
	25,000-50,000	3	2	—
	50,000-75,000	5	4	—
	75,000-100,000	8	7	—
	100,000-150,000	7	1	—
	150,000-200,000	1	2	—
	>200,000	1	0	—
**Town or city population range**				
	1000-5000	1	1	—
	6000-15,000	4	2	—
	16,000-30,000	2	2	—
	31,000-60,000	8	2	—
	61,000-100,000	1	1	—
	101,000-250,000	6	1	—
	251,000-500,000	2	4	—
	501,000-750,000	1	1	—
	>750,000	2	4	—
**Education**				
	Some college without obtaining a degree	4	N/A^b^	N/A^b^
	2-year degree	6	N/A^b^	N/A^b^
	Bachelor’s degree	8	N/A^b^	N/A^b^
	Graduate degree	9	N/A^b^	N/A^b^

^a^These veterans did not participate as panelists; only their spouses participated.

^b^The gender of the spouse’s veteran partner, as well as the education level of spouses and their veteran partners were not collected.

^c^Due to a data collection error, the panelists’ gender was not recorded at the time of round 1 panels, although it was collected during rounds 2 and 3. Thus, the gender of the 3 veteran panelists and 3 spouse panelists who did not return after round 1 panels was unavailable.

^d^N/A: not applicable.

^e^As spouses shared a household with their Veteran spouse, they both had the same total household income and town or city population size.

### Qualitative Feedback on PTBS Content and Design

The panelist feedback relevant to each user-engagement-influencing construct [36] is summarized below. The italicized block quotes are verbatim transcriptions of relevant panelist comments.

### User-Related Constructs and Associated Panelist Feedback

#### User Beliefs

Previous research has found that users’ beliefs can promote or impair engagement with digital MH interventions [36]. For example, increased user engagement has been found when users believe that they have mental health symptoms, that these symptoms are worth addressing, and that the intervention can help them address those symptoms.

On several occasions, the panelists provided feedback on user engagement–associated beliefs. Spouses reported that they personally played an important role in their veterans’ realization that they needed to change existing maladaptive behaviors. Indeed, spouses noted that they often initiated conversations about their veterans’ mental health symptoms:

No- he usually doesn’t say anything, but I can tell when he looks sleep deprived or I’ve woken up and he’s not in bed, and so I can figure out that he’s once again having problems sleeping.

When asked a multiple-choice question about what they did to prepare to discuss how their veteran could treat their mental health symptoms, the spouses’ most frequent response was that they would research resources on the web themselves before sharing them with their veteran:

I would probably do a little research on it and get more familiar on what it is, the course or the online class. What it’s about and how it would help him, I would look at the issues they target. I would want to know how it would help him.

Having clear motivations for behavioral change and the belief that an intervention will facilitate that change is important for user engagement [40,41]. Panelists noted that a description of the negative effects of chronic insomnia would be particularly motivating to veterans, as it would persuade them that insomnia is an important problem that needs to be addressed. To identify other common motivations for change that could be emphasized within PTBS, veteran panelists were asked about what motivated them to make considerable behavioral changes in the past. Their prior motivations fell into several themes, as shown in Table 2 along with representative panelist quotes.

**Table 2:** Themes in motivation for behavior change reported by veteran panelists and representative quotes.

Motivational theme and description			Representative veteran quotes
**Self-care**			
	The realization that something has gone off-track in regard to the Veteran’s health and the desire to get back to a healthier state	“I realized I couldn’t keep up with my kids for the first time.” “I looked in the mirror and didn’t recognize myself.” “I didn’t like how I felt or looked, so that drove me to start exercising every single day and eat a lot healthier.”	
**Loved ones**			
	The desire to be an example for their loved ones and to be around to support their children or grandchildren	“I quit smoking 5 years ago…. We adopted 2 kids and I have grandkids. Just making sure I was around to see them grow up and wanted them to have me around.”	
**Financial or career advancement**			
	Anticipation of a benefit to one’s finances or career	“Was missing that degree checkbox on my resume. Lack of hire-ability for mobility from my current position.” “I quit smoking 20 years ago. What prompted me was how much money was going out.”	
**Personal identity**			
	The sense that the behavior change is the next logical step in their life or is consistent with who they want to become in life	“I always wanted to teach, so I did my Master’s… and I’m now a substitute teacher.” “After I got out of the Marines, I decided to go back to school for a graduate degree. It was the logical choice, always something I was interested in and I had GI Bill funding.”	

#### Mental Health and Technology Literacy and Experiences

Prior studies have found that digital MH intervention users’ preexisting literacy and experiences with traditional MH services and digital MH technologies affect user engagement, with increased literacy and positive experiences associated with greater engagement [36]. Overall, most veteran panelists reported having had positive prior experience using web-based self-guided materials for learning, growth, or behavioral change. Most veteran panelists also reported feeling confident that they would succeed when starting to use a new web-based resource.

#### Integration Into Life

Prior studies have found that users’ ability to find time and space in their regular routine to work on a digital MH intervention is associated with increased user engagement [36].

One way in which PTBS attempts to convey its manageability and convenience is by visually displaying the users’ progress through the course. At regular intervals throughout PTBS, users are shown a graphic representation of how much of the course they have completed in the form of a stylized road map: as the user progresses through the course, a vehicle is depicted traveling down a road. When the vehicle reaches the end of the road, the user has completed the course. When shown this graphic, a large portion of panelists reported that clear and repeated updates on their progress through the course would motivate them to complete the course:

I like the visual. I like that I can see a beginning and an end. My [Veteran] husband would like it too because it’s a picture that he can visually see where he’s at in the process.

I like using a tracking system to keep track of what I'm doing so that I can look and see “okay, last time, you did this, so you can do a little bit more this time.”

Both veterans and spouses consistently stated that veterans were more likely to start and complete self-help materials that did not appear to require a large investment in time or effort. When panelists were asked what features they found desirable in digital self-help programs, the most commonly mentioned were those that made the programs easier to do, such as reminder emails, the ability to complete the course in many short work sessions, and the ability to quickly resume where one left off when logging back into the program.

Perhaps the most striking illustration of the importance of conveying PTBS’s ability to readily integrate into users’ lives came from the 3 panelists who self-reported current or prior problems with insomnia. After seeing an initial prototype of the PTBS sleep diary, only 1 of the 3 panelists reported that they would be interested in using it to address insomnia. The other 2 panelists said that they would recommend the sleep diary to other individuals with insomnia symptoms; however, they personally felt that the sleep diary in the course prototype required too much work for them to use regularly.

### Program-Related Constructs and Associated Panelist Feedback

#### Type of Content

Prior studies have shown that user engagement with digital MH interventions is facilitated when the content of the intervention is perceived as credible and when the users report greater satisfaction with the features, content, and modality of the intervention [36].

On several occasions, panelists reported that trustworthy reviews and endorsements were an important factor they considered when deciding whether to use a self-help program on the web. Panelists found the following items particularly persuasive when deciding whether to use a particular program: (1) unbiased reviews, such as those found in a smartphone app store; (2) recorded reviews or testimonials by veterans or individuals in the military; and (3) personal recommendations from military service members, veterans, employers, family, trusted friends, or their health care providers:

I read the reviews. How good that particular program is for other people in general. I delve into their track record.

VA locally mentioned the [mental health] application, so I went and looked it up… Worked really well.

#### Perceived Fit

Prior studies have shown that user engagement is facilitated when users feel that the intervention is consistent with their culture and values, relevant to their lived experience, and personalized to their particular needs [36]. Elements found to promote an intervention’s perceived fit include having the people presented in the intervention be those the user can identify with, using culturally appropriate content, using text at a suitable reading level, eliminating information irrelevant to the user’s life situation, and limiting the use of technical language or jargon.

Consistent with prior studies of perceived fit, several veterans described a preference for materials or programs explicitly geared toward veterans or members of the military:

[A particular online school program] dominated because they had a military social work program and with the resources they had for Veterans, it seemed like it was meant to be.

Spouse panelists voiced frustration with previous self-help materials they had received (eg, pamphlets on how to help their veteran readjust following deployment). They felt that these materials provided an overly rosy description of the issues they faced.

Most panelists reported positive impressions of video testimonials by veterans who had completed CBT-I treatment. However, when reading some of the written testimonials that appeared early in the course, a few panelists reported that the language seemed overly scripted and even questioned the authenticity of the written testimonials:

They don’t look real. Just look like some key words that people made up and put under some random pictures. The quotes themselves. “It changed my life and saved my career” just seems ridiculous. Not real.

The most frequent and emphatic feedback provided by the panelists was to reduce the amount of text in the course and to make the remaining text as clear and straightforward as possible. The panelists praised succinct and direct language, such as a description of whom the course was intended for:

Draws my eye to the initial caption ‘can’t fall asleep, can’t stay asleep.’ It would interest me from the very beginning because it shows the very core of the issue, so that’s where my eye would go first.

Panelists repeatedly recommended that all unnecessary details be trimmed from the content and only the core concepts be communicated to the user in a concise and clear way:

Easy to understand, but it’s a wall of text. I don’t like the wall of texts and feel like most veterans would skip reading since death by PowerPoint.

In response to a lengthy paragraph describing how the information collected during the course was stored and how user privacy was protected, a panelist commented:

The text could have been summarized in one sentence: Stored confidentially and securely. Secure is important. Then the lock picture would reinforce that.

Some of the most consistently praised elements in PTBS were illustrations and diagrams that clarified PTBS concepts. Conversely, some of the most criticized elements were illustrations and diagrams, whose meaning was unclear or required extensive explanation.

#### Perceived Usefulness

Previous studies have found that users’ perception that a digital MH intervention is useful facilitates user engagement [36]. Intervention features found to facilitate perceived usefulness include being able to understand the data presented in the intervention, having a clear sense of what action the user needs to take, and conveying that the intervention has clear advantages over other care that the user has received.

Panelists emphasized the importance of clearly communicating CBT-I’s efficacy in treating chronic insomnia. Panelists suggested that PTBS materials explicitly describe the evidence for the better efficacy of CBT-I than other interventions for treating chronic insomnia that require less time and effort but have less evidence for their efficacy (eg, the use of a wrist-mounted sleep-tracking device).

### Evaluation Panel Feedback Relevant to Technology and Environment–Related Constructs: Technology

Previous studies have found that the technology used to deliver a digital MH intervention can impact user engagement [36]. Technical issues with the intervention, including crashes, bugs, or poor usability, have been found to negatively impact user engagement. Conversely, technologies that facilitate the use of an intervention where and when it is convenient have been found to increase user engagement.

With regard to facilitating convenient access to the intervention, multiple panelists reported that they wanted to access PTBS content via a smartphone app as opposed to a website. In particular, panelists said they wanted to use a smartphone app to fill in their sleep diary, because they would need to do this daily and potentially immediately upon waking:

If I have insomnia, I’m already tired and cranky and I don’t want to look at all these buttons. There’s a lot of steps. And it’s a lot to remember right when you’re waking up. It’s a lot, and if I have to go my computer and open it up and put down the information.

### Intervention Design Choices Informed by the Panelists’ Feedback

**Figure 2:**
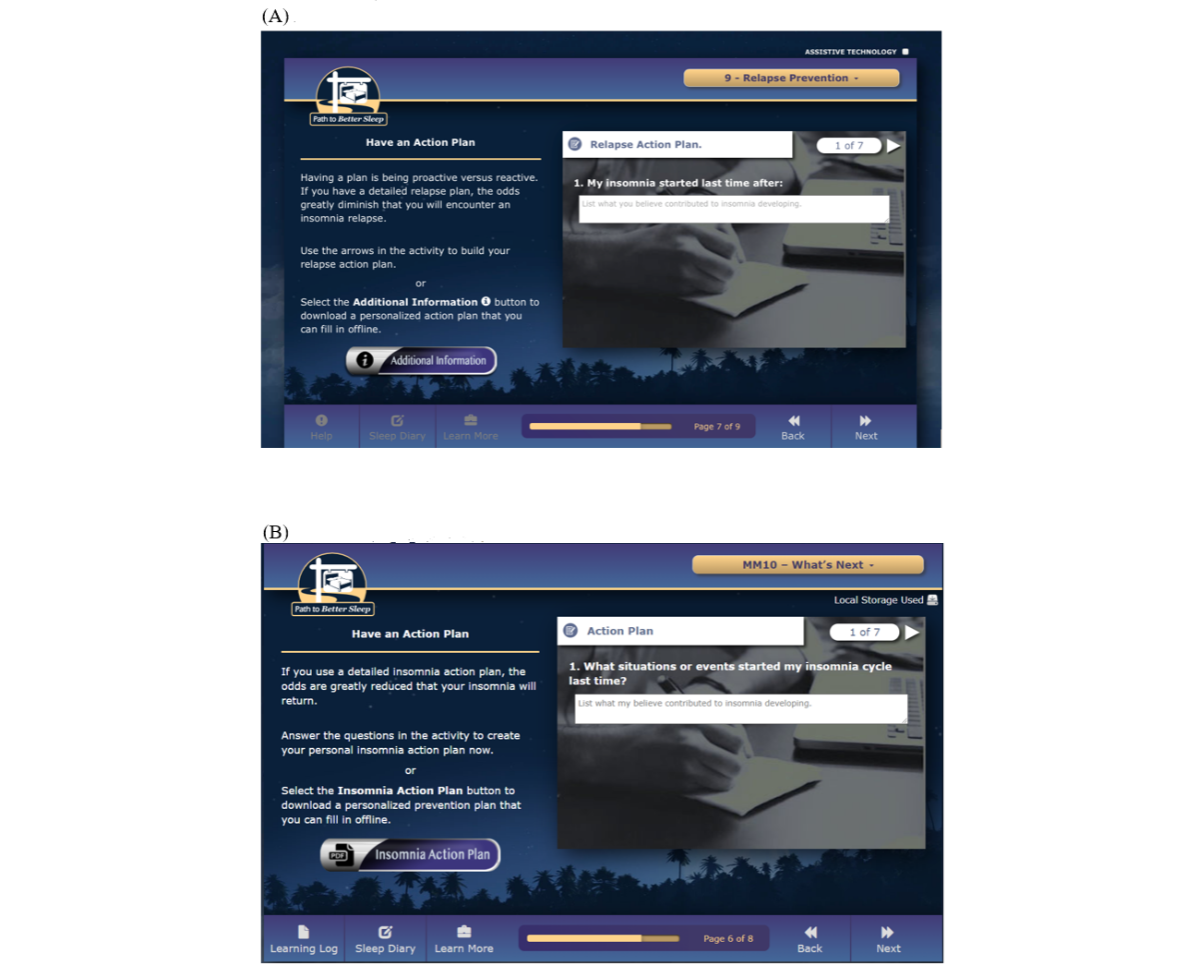
An example of a draft version of Path to Better Sleep content and its revised version following panelist feedback. (A) Initial draft presented to panelists for feedback. For the panelists, the word “relapse” had a negative connotation of substance addiction and of blaming the veteran for the recurrence of symptoms. (B) Revised draft following panelist feedback. Revisions made based on panelist feedback to this page and others. The word “relapse” was removed; the amount of text was reduced; user instructions were more accurately specified (ie, “Answer the questions in the activity to create your personal insomnia action plan” instead of “Use the arrows in the activity to build your relapse action plan”); and the more informative label “Insomnia Action Plan” was placed on the “Additional Information” button.

The feedback provided by the veteran and spouse panelists informed the design and development of PTBS. Revisions made in response to feedback included those made to address feedback on specific pieces of content (eg, not using the word *relapse* to describe symptom recurrence, as *relapse* was perceived as stigmatizing by several panelists) as well as changes that involved large portions of the intervention (eg, revising text throughout the intervention to make it concise and straightforward as possible). Examples of intervention design choices informed by panelist feedback are shown in Textbox 3. An example of a draft version of PTBS content and its revised version, following the implementation of panelist feedback, is shown in Figure 2.

**Intervention design decisions that were informed by panelists’ feedback organized by the user engagement construct they relate to.**

**Construct:** **user beliefs**

Feedback received

- Spouses are key influencers in the development of veteran beliefs regarding mental health symptoms and interventions.

Design choices

- Made the course freely available to anyone on the internet, thus allowing spouses to research and explore the course themselves.
- In the design of the communications campaign to disseminate Path to Better Sleep (PTBS), included direct messaging to spouses of veterans.

Feedback received

- Veterans’ motivations for prior efforts at behavioral change frequently fell into 4 common themes: self-care, loved ones, financial or career advancement, and personal identity.

Design choices

- Selected veteran testimonials that discussed motivations for addressing insomnia symptoms that aligned with common motivational themes.

**Construct: perceived fit**

Feedback received

- Both veterans and spouses voiced distrust of testimonials and other content they perceived as presenting unrealistic or overly positive descriptions of veterans’ challenges or the effectiveness of the intervention.

Design choices

- Selected veteran testimonial videos where veterans provided detailed descriptions of the challenges caused by chronic insomnia, discussed their initial ambivalence about whether cognitive behavioral therapy for insomnia (CBT-I) could help them, reported that CBT-I required significant effort on their part, and concluded that CBT-I’s treatment benefits were worth the effort.

Feedback received

- Make all intervention content, especially text, as clear, succinct, and straightforward as possible.

Design choices

- Made revisions throughout the course to make text and diagrams more concise, clear, and succinct.

**Construct: perceived usefulness**

Feedback received

- Explain the effectiveness of CBT-I techniques to justify the greater time and effort they require compared with other less effective self-help strategies (such as the use of sleep-tracking fitness devices that purport to improve sleep quality).

Design choices

- Emphasized evidence for the efficacy of CBT-I techniques compared with other self-help strategies.

**Construct: technology**

Feedback received

- The features most valued in previously used self-help resources were those that made them easy to use; the PTBS sleep diary needs to be accessible via a smartphone app and easy to fill out.

Design choices

- Made it easy for users to jump to any module within the course so that users could quickly resume where they left off.
- Integrated the use of “CBT-I Coach” into the course. CBT-I Coach is a Veterans Affairs–created mobile app that includes a sleep diary feature, along with summaries of key CBT-I concepts and other convenient features.

## Discussion

### Principal Findings

Veteran and spouse evaluation panels commissioned during the development of PTBS provided valuable qualitative feedback that guided revisions to PTBS’s content and design with the goal of improving eventual user engagement. Furthermore, the panelists’ feedback was consistent with previous qualitative and quantitative studies on the factors that influence user engagement with digital MH interventions.

To increase veterans’ motivation to complete PTBS, evaluation panelists recommended that we emphasize evidence for the efficacy of the CBT-I techniques taught in PTBS (eg, sleep scheduling and stimulus control), especially in comparison with other self-help methods that purport to improve sleep while requiring less time or effort (eg, using a fitness tracker to track sleep patterns). The panelists’ feedback aligns with previous research showing that treatment credibility and expectancy (elements of the perceived usefulness construct) are positively correlated with sustained interactions with digital MH interventions [42-47]. The importance of perceived usefulness for user engagement has also been shown in a study of individuals who preferred face-to-face psychotherapy to digital psychotherapy: this preference was associated with the belief that face-to-face psychotherapy is more effective than digital MH interventions [48]. This suggests the importance of educating users about research suggesting that dCBT-I has similar efficacy as traditional CBT-I [27].

In line with our panelists’ repeated admonishment to clarify and simplify course content, previous research has shown that digital MH intervention users are more likely to stop using an intervention when they feel that it is difficult or stressful to use [47,49-52]. Even negative experiences with a single component of a digital MH intervention may decrease the rate of sustained interaction with the intervention [50,51]. Unrealistic or irrelevant content may also decrease user engagement. For example, our spouse panelists noted their dissatisfaction with excessively sanguine self-help materials: they concluded that these self-help materials were unrealistic and thus could not be helpful. Consistent with their report, studies have shown that a digital MH intervention’s perceived irrelevance to its users’ life experiences is associated with reduced rates of sustained interaction [49,50]. Another way to increase sustained interaction by veteran users may be to remind them of factors that motivated behavioral change in the past. Prior research has found that motivation for change increases adherence to digital MH interventions [40], including adherence to dCBT-I in particular [41].

It is important to acknowledge the limitations of the feedback gathered from the veteran and spouse evaluation panels. The panels’ facilitators were contracted by the VA to provide feedback on the materials; these facilitators used a prewritten script of questions and discussion prompts during their facilitation of the evaluation panels. Thus, panelists were likely to provide feedback only on materials and topics that the VA team had preidentified as requiring review and feedback. Thus, there may have been aspects of the intervention that panelists could have provided important feedback on but that were not presented to them for feedback. In addition, while efforts were taken to minimize the bias of the feedback provided by the panelists (eg, using open-ended questions wherever possible and referring to participants only by their first name and city), feedback from group evaluation panels is inherently susceptible to several validity issues, including panelist social dynamics (eg, dominance and cohesion) and moderator biasing [53].

### Recommendations for Other Digital MH Intervention Developers

The lessons learned from our use of qualitative evaluation panels may apply to the development of other digital MH interventions. First, we suggest that conducting qualitative evaluation panels during digital MH intervention development is a useful practice. PTBS’s evaluation panels helped to highlight areas where the course content was insufficiently tailored to veterans’ needs and, therefore, likely to reduce user engagement.

A second lesson that might be useful to other digital MH intervention developers is on the importance of soliciting input from stakeholders who facilitate successful use of digital MH interventions by their target audience. The spouse panelists’ feedback supported our intuition that veterans’ spouses play an important role in facilitating their use of digital self-help materials. This spouse feedback supported design and dissemination decisions aimed at helping spouses facilitate PTBS’s use by their veteran partners; for example, the targeting of some dissemination materials toward veterans’ family members rather than the veterans themselves. Digital MH interventions targeting other populations may also benefit from identifying stakeholders who facilitate uptake by their target users. For example, the developer of a digital MH intervention for use in employee assistance programs might conduct content evaluation panels with human resource managers, as these managers are likely to be essential in the adoption and use of their intervention by employees.

### Conclusions

In this manuscript, we describe qualitative evaluation panels conducted during the development of PTBS, a dCBT-I intervention commissioned by the Department of Veterans Affairs and freely available on the web to veterans and members of the public [30]. Evaluation panels comprising veterans and spouses of veterans provided feedback that guided revisions to PTBS during its development.

## Appendix

Multimedia Appendix 1 Facilitator guide for round 1 of veteran panels.

Multimedia Appendix 2 Facilitator for round 1 of spouse panels.

Multimedia Appendix 3 Facilitator guide for round 2 of veteran panels.

Multimedia Appendix 4 Facilitator guide for round 2 of spouse panels.

Multimedia Appendix 5 Facilitator guide for round 3 of veteran panels.

Multimedia Appendix 6 Facilitator guide for round 3 of spouse panels.

## Abbreviations

CBT-I: cognitive behavioral therapy for insomnia
dCBT-I: digital cognitive behavioral therapy for insomnia
MH: mental health
PTBS: Path to Better Sleep
VA: veterans affairs
VHA: Veterans Health Administration

## References

[1] National Institutes of Health (2005). National institutes of health state of the science conference statement on manifestations and management of chronic insomnia in adults, June 13-15, 2005. Sleep.

[2] Colvonen PJ, Almklov E, Tripp JC, Ulmer CS, Pittman JO, Afari N (2020). Prevalence rates and correlates of insomnia disorder in post-9/11 veterans enrolling in VA healthcare. Sleep.

[3] Pigeon WR, Pinquart M, Conner K (2012). Meta-analysis of sleep disturbance and suicidal thoughts and behaviors. J Clin Psychiatry.

[4] Hom MA, Lim IC, Stanley IH, Chiurliza B, Podlogar MC, Michaels MS, Buchman-Schmitt JM, Silva C, Ribeiro JD, Joiner Jr TE (2016). Insomnia brings soldiers into mental health treatment, predicts treatment engagement, and outperforms other suicide-related symptoms as a predictor of major depressive episodes. J Psychiatr Res.

[5] Hertenstein E, Feige B, Gmeiner T, Kienzler C, Spiegelhalder K, Johann A, Jansson-Fröjmark M, Palagini L, Rücker G, Riemann D, Baglioni C (2019). Insomnia as a predictor of mental disorders: a systematic review and meta-analysis. Sleep Med Rev.

[6] Vgontzas AN, Fernandez-Mendoza J, Liao D, Bixler EO (2013). Insomnia with objective short sleep duration: the most biologically severe phenotype of the disorder. Sleep Med Rev.

[7] Wardle-Pinkston S, Slavish DC, Taylor DJ (2019). Insomnia and cognitive performance: a systematic review and meta-analysis. Sleep Med Rev.

[8] Kucharczyk ER, Morgan K, Hall AP (2012). The occupational impact of sleep quality and insomnia symptoms. Sleep Med Rev.

[9] Kyle SD, Morgan K, Espie CA (2010). Insomnia and health-related quality of life. Sleep Med Rev.

[10] Irish LA, Kline CE, Gunn HE, Buysse DJ, Hall MH (2015). The role of sleep hygiene in promoting public health: a review of empirical evidence. Sleep Med Rev.

[11] Riemann D, Baglioni C, Bassetti C, Bjorvatn B, Dolenc Groselj L, Ellis JG, Espie CA, Garcia-Borreguero D, Gjerstad M, Gonçalves M, Hertenstein E, Jansson-Fröjmark M, Jennum PJ, Leger D, Nissen C, Parrino L, Paunio T, Pevernagie D, Verbraecken J, Weeß HG, Wichniak A, Zavalko I, Arnardottir ES, Deleanu OC, Strazisar B, Zoetmulder M, Spiegelhalder K (2017). European guideline for the diagnosis and treatment of insomnia. J Sleep Res.

[12] Schroeck JL, Ford J, Conway EL, Kurtzhalts KE, Gee ME, Vollmer KA, Mergenhagen KA (2016). Review of safety and efficacy of sleep medicines in older adults. Clin Ther.

[13] Trauer JM, Qian MY, Doyle JS, Rajaratnam SM, Cunnington D (2015). Cognitive behavioral therapy for chronic insomnia: a systematic review and meta-analysis. Ann Intern Med.

[14] Talbot LS, Maguen S, Metzler TJ, Schmitz M, McCaslin SE, Richards A, Perlis ML, Posner DA, Weiss B, Ruoff L, Varbel J, Neylan TC (2014). Cognitive behavioral therapy for insomnia in posttraumatic stress disorder: a randomized controlled trial. Sleep.

[15] Ho FY, Chan CS, Tang KN (2016). Cognitive-behavioral therapy for sleep disturbances in treating posttraumatic stress disorder symptoms: a meta-analysis of randomized controlled trials. Clin Psychol Rev.

[16] Belleville G, Cousineau H, Levrier K, St-Pierre-Delorme MÈ (2011). Meta-analytic review of the impact of cognitive-behavior therapy for insomnia on concomitant anxiety. Clin Psychol Rev.

[17] Gebara MA, Siripong N, DiNapoli EA, Maree RD, Germain A, Reynolds CF, Kasckow JW, Weiss PM, Karp JF (2018). Effect of insomnia treatments on depression: a systematic review and meta-analysis. Depress Anxiety.

[18] Schutte-Rodin S, Broch L, Buysse D, Dorsey C, Sateia M (2008). Clinical guideline for the evaluation and management of chronic insomnia in adults. J Clin Sleep Med.

[19] The Management of Chronic Insomnia Disorder and Obstructive Sleep Apnea Work Group, The Office of Quality, Safety and Value, VA, Washington, DC, Office of Evidence Based Practice, U.S. Army Medical Command (2019). VA/DoD clinical practice guideline for the management of chronic insomnia disorder and obstructive sleep apnea. Department of Veterans Affairs, Department of Defense.

[20] Manber R, Carney C, Edinger J, Epstein D, Friedman L, Haynes PL, Karlin BE, Pigeon W, Siebern AT, Trockel M (2012). Dissemination of CBTI to the non-sleep specialist: protocol development and training issues. J Clin Sleep Med.

[21] Karlin BE, Trockel M, Taylor CB, Gimeno J, Manber R (2013). National dissemination of cognitive behavioral therapy for insomnia in veterans: therapist- and patient-level outcomes. J Consult Clin Psychol.

[22] Koffel E, Bramoweth AD, Ulmer CS (2018). Increasing access to and utilization of cognitive behavioral therapy for insomnia (CBT-I): a narrative review. J Gen Intern Med.

[23] Farmer CM, Hosek SD, Adamson DM (2016). Balancing demand and supply for veterans' health care: a summary of three RAND assessments conducted under the veterans choice act. Rand Health Q.

[24] National Academies of Sciences, Engineering, and Medicine (2018). Department of Veterans Affairs mental health services: need, usage, and access and barriers to care. Evaluation of the Department of Veterans Affairs Mental Health Services.

[25] Andrews G, Basu A, Cuijpers P, Craske MG, McEvoy P, English CL, Newby JM (2018). Computer therapy for the anxiety and depression disorders is effective, acceptable and practical health care: an updated meta-analysis. J Anxiety Disord.

[26] Andersson G, Titov N (2014). Advantages and limitations of internet-based interventions for common mental disorders. World Psychiatry.

[27] Soh HL, Ho RC, Ho CS, Tam WW (2020). Efficacy of digital cognitive behavioural therapy for insomnia: a meta-analysis of randomised controlled trials. Sleep Med.

[28] Taylor DJ, Peterson AL, Pruiksma KE, Young-McCaughan S, Nicholson K, Mintz J, STRONG STAR Consortium (2017). Internet and in-person cognitive behavioral therapy for insomnia in military personnel: a randomized clinical trial. Sleep.

[29] Veteran training. U.S. Department of Veterans Affairs.

[30] Greene CJ, Ulmer CS, Farrell-Carnahan L, Mackintosh M (2017). Path to better sleep course. U.S. Department of Veterans Affairs.

[31] Ulmer CS, Farrell-Carnahan L, Hughes JM, Manber R, Leggett MK, Tatum J, Swinkels C, Beckham JC, Mid-Atlantic (VISN 6) Mental Illness Research, Education and Clinical Center (MIRECC) (2018). Improve your sleep: a self-guided approach for veterans with insomnia (Self-Help Workbook). U.S. Department of Veterans Affairs.

[32] Yardley L, Morrison L, Bradbury K, Muller I (2015). The person-based approach to intervention development: application to digital health-related behavior change interventions. J Med Internet Res.

[33] van Gemert-Pijnen JE, Nijland N, van Limburg M, Ossebaard HC, Kelders SM, Eysenbach G, Seydel ER (2011). A holistic framework to improve the uptake and impact of eHealth technologies. J Med Internet Res.

[34] Karpathakis K, Libow G, Potts HW, Dixon S, Greaves F, Murray E (2021). An evaluation service for digital public health interventions: user-centered design approach. J Med Internet Res.

[35] Maramba I, Chatterjee A, Newman C (2019). Methods of usability testing in the development of eHealth applications: a scoping review. Int J Med Inform.

[36] Borghouts J, Eikey E, Mark G, De Leon C, Schueller SM, Schneider M, Stadnick N, Zheng K, Mukamel D, Sorkin DH (2021). Barriers to and facilitators of user engagement with digital mental health interventions: systematic review. J Med Internet Res.

[37] Donkin L, Christensen H, Naismith SL, Neal B, Hickie IB, Glozier N (2011). A systematic review of the impact of adherence on the effectiveness of e-therapies. J Med Internet Res.

[38] Millward LJ, Breakwell GM, Smith JA, Wright DB (2012). Focus groups. Research Methods in Psychology. 4th edition.

[39] Erbes CR, Kuhn E, Polusny MA, Ruzek JI, Spoont M, Meis LA, Gifford E, Weingardt KR, Campbell EH, Oleson H, Taylor BC (2020). A pilot trial of online training for family well-being and veteran treatment initiation for PTSD. Mil Med.

[40] Farrer LM, Griffiths KM, Christensen H, Mackinnon AJ, Batterham PJ (2013). Predictors of adherence and outcome in internet-based cognitive behavior therapy delivered in a telephone counseling setting. Cogn Ther Res.

[41] Hebert EA, Vincent N, Lewycky S, Walsh K (2010). Attrition and adherence in the online treatment of chronic insomnia. Behav Sleep Med.

[42] Hasson H, Brown C, Hasson D (2010). Factors associated with high use of a workplace web-based stress management program in a randomized controlled intervention study. Health Educ Res.

[43] Geraghty AW, Wood AM, Hyland ME (2010). Attrition from self-directed interventions: investigating the relationship between psychological predictors, intervention content and dropout from a body dissatisfaction intervention. Soc Sci Med.

[44] Boettcher J, Renneberg B, Berger T (2013). Patient expectations in internet-based self-help for social anxiety. Cogn Behav Ther.

[45] Cavanagh K, Shapiro DA, Van Den Berg S, Swain S, Barkham M, Proudfoot J (2009). The acceptability of computer-aided cognitive behavioural therapy: a pragmatic study. Cogn Behav Ther.

[46] Nordgreen T, Havik OE, Ost LG, Furmark T, Carlbring P, Andersson G (2012). Outcome predictors in guided and unguided self-help for social anxiety disorder. Behav Res Ther.

[47] Berman MI, Buckey Jr JC, Hull JG, Linardatos E, Song SL, McLellan RK, Hegel MT (2014). Feasibility study of an interactive multimedia electronic problem solving treatment program for depression: a preliminary uncontrolled trial. Behav Ther.

[48] Renn BN, Hoeft TJ, Lee HS, Bauer AM, Areán PA (2019). Preference for in-person psychotherapy versus digital psychotherapy options for depression: survey of adults in the U.S. NPJ Digit Med.

[49] Price M, Gros DF, McCauley JL, Gros KS, Ruggiero KJ (2012). Nonuse and dropout attrition for a web-based mental health intervention delivered in a post-disaster context. Psychiatry.

[50] Gerhards SA, Abma TA, Arntz A, de Graaf LE, Evers SM, Huibers MJ, Widdershoven GA (2011). Improving adherence and effectiveness of computerised cognitive behavioural therapy without support for depression: a qualitative study on patient experiences. J Affect Disord.

[51] Schneider J, Sarrami Foroushani P, Grime P, Thornicroft G (2014). Acceptability of online self-help to people with depression: users' views of MoodGYM versus informational websites. J Med Internet Res.

[52] Scott K, Beatty L (2013). Feasibility study of a self-guided cognitive behaviour therapy internet intervention for cancer carers. Aust J Prim Health.

[53] Stewart DW, Shamdasani PN (2015). Focus Groups: Theory and Practice. 3rd edition.

