# Supplementary material for: The Use of Evaluation Panels During the Development of a Digital Intervention for Veterans Based on Cognitive Behavioral Therapy for Insomnia: Qualitative Evaluation Study
Source: JMIR Form Res. 2023 Mar 6;7:e40104. doi: 10.2196/40104 (PMC10028512; doi:10.2196/40104)
Supplement: Multimedia Appendix 1 [file formative_v7i1e40104_app1.docx]

U.S. Department of Veterans Affairs –

Mental Health Services

## Veterans Panels – Round 1

## Moderator Guide

## MARCH 2017

Research OBJECTIVES:

1. Gather feedback from Veterans on the perceived motivators, facilitators and barriers to uptake and persistence with online “self help” resources.
2. Gather feedback from Veterans on their decision-making and follow-through when making changes in their lives.

#### SCHEDULE

| Date | Time (EST) |
| --- | --- |
| Tuesday, March 14, 2017 | 4:00 – 5:00 pm EST |
| Wednesday, March 15, 2017 | 6:00 – 7:00 pm EST |
| Thursday, March 16, 2017 | 8:00 – 9:00 pm EST |

# INTRODUCTION TO PANEL (10 minutes)

Thank you for joining us today. I’m [Facilitator] and I’ll be moderating today’s discussion.

Today’s discussion is designed to get feedback from Veterans, such as yourself, in order to help the Department of Veterans Affairs (which we will refer to as VA) better understand where you get information about health issues and to improve the technologies and services that VA provides to Veterans.

Apart from some general questions, we’ll also talk about your thoughts on “self help” tools as well as talking about some of the ways that all of you have brought about changes in your life and the things that motivated you and kept you going. I’m really looking forward to hearing all of your opinions and feedback!

As we move through the discussion today, I’d like you to be open and honest. There are no right or wrong answers. It’s okay to disagree with one another; I want to hear a range of opinions, so please do speak freely. Please just remember to respect the opinions of others in the group.

Please remember that any information collected will be kept private, no one outside of this meeting will know who said what – your personal identities will not be shared with VA. This session is being recorded, but for our analysis only.

I may sometimes ask you to write down your thoughts prior to sharing them with the group, so please do have a pen and paper handy.

We have a lot of ground to cover, so I may jump around at times or not call on everyone for each question. If you have something to add please just chime in or use the chat feature. We want this to be a conversation!

My colleague, [Facilitator], who you spoke to for your recent technical check, is going to do a quick review of a few technical features before we begin. We will also do a roll call and hear a little bit about each of you.

With that, let’s get started.

**Technical Review by [**Facilitator]**.**

**Back to [**Facilitator]**for Roll Call:** To kick things off, I’d like each of you to provide a quick introduction: give us your first name, how long you served and what branch, where you live now, and something significant going on in your life right now. Nothing too personal, just a little about yourself.

(Moderator – **[**Facilitator]*will briefly reintroduce herself in this format to give respondents an idea of what they should say. Then,* **[**Facilitator]*to call out respondents one by one, by first name. Prompt each individual attendee to provide their introduction.*)

Thank you all for those introductions.

OK, I think we’re ready to get started.

# PART 1: intrinsic & extrinsic motivators and perceived barriers to use of online courses and “SELF HELP” (25 minutes)

****Content in parentheses is not to be spoken aloud, but rather used as a guide to rephrase questions if they are not understood by participants****

***So to start, I’d like for us to talk a bit about online courses and online “self help” resources that you may have used…***

1. **With all of the online resources available to us these days, I’d like to understand what types of online resources you have used for learning, growth or just general “self help”. What are some of the general topics you’ve explored with online learning?** (ie: *Online college or extension courses; online fitness or nutrition programs; online stress relief/meditation; mental health/therapy programs, etc.*)
2. *****Use the Adobe Connect polling feature*** I’d like to take a quick poll here. When it comes to seeking out online resources (such as the classes and “self help” we’ve just discussed), which phrase fits YOUR personal style:**
   1. **I need help with something. I find the resources I need and just do it.**
   2. **I need help with something, I find the resource, and I bookmark it to look into…one day.**
   3. **I know I need help with something, but with one thing and another, I never seem to have time to find the resources, let alone commit to an online class.**
   4. **I don’t need help. But my spouse/partner/friend keeps pestering me, so I’ll just do it.**
   5. **Help? Who needs help? I figure things out for myself.**
3. **So, again referring to the online learning or “self help” resources that each of you has used, how did you find those resources?** (ie: *online search, spouse, friend, doctor, etc*.)
   1. *If anyone references OFFLINE*: **For those who found the resources OFFLINE** (ie: paper materials- posters/flyers/brochures, referrals, word of mouth…)**, did you have any trouble later finding the website online when you decided to look for it?**
   2. **For those who found the resource online, how specifically did you come across it**? (i.e.: Simple google search; followed a link from another trusted website; read about it in an online news article; saw it mentioned by a group I follow on facebook/twitter, etc.)
4. **What drew you to that particular resource vs. others that were available?** (ie: *near top of online search*, *free or low cost; user friendly; looked interesting; trusted the organization offering it; came recommended by someone I trust, etc*.)
5. **Before settling on that program, did you “window shop” by signing up for or trying out other similar resources?**
   1. **If so, can you recall what turned you off about the resources you decided NOT to use?**
   2. **What was the difference between the ones you didn’t choose and the resource you DID choose?**
6. **In regards to the resource you did choose in the end, did you complete the entire online class or program that you began?**
7. **If so, about how long did it take you?** (nb; *If people mention days or weeks, try to clarify how much time PER day or week they devoted to the resource* *so we can compare time in HOURS*)
   1. **What personality traits or qualities of yours do you think helped you**? (*ie: “stubbornness”, fast learner, grit, determination, noticed progress, etc*.)
   2. **What features of the program or outside of yourself helped you?** (*ie: it was quick; enjoyable; reminder e-mails/texts/phone calls; encouragement from a loved one; check-in with doctor, boss, or peer; flexibility/ability to control when to engage with the program, etc.)*
8. **If you didn’t complete the online class or program, how long did you spend on it before stopping?**
   1. **What are some of the reasons you stopped**? (*ie: took too much time; just forgot; didn’t seem to be helpful; not useful; not enjoyable; I spend too much time in front of a computer at work as it is…)*
   2. **In hindsight, what do you think might have helped you to keep going or finish?** (ie: *quicker/shorter version of the resource; reminder e-mails/texts/calls; encouragement from someone close; follow-up or check-in with doctor, boss or peer (accountability), more flexibility/ability to control when to engage with the program, reminders of progress/incremental goals; more tailored program, etc.)*
9. *****Use the Adobe Connect polling feature*** I’d like to take a quick poll here. If you had to choose one phrase that expresses your general attitude when beginning a new online learning or “self help” resource (like any of the ones we just talked about), which of these would it be?**
   1. **“I’ve got this. Bring it on.”**
   2. **“I think I can do this. I’ll give it a try.”**
   3. **“I’m not sure I can do this, but it’s worth a shot.”**
   4. **“There’s no way this is going to work, but I’ll try.”**
   5. **“What a waste of time. Here goes nothing.”**

# PART 2: decision-making and follow-through (20 minutes)

***Thank you so much for all of that great feedback. Now we’re going to talk a bit more broadly about your experiences with implementing changes in your life.***

1. **I’d like you to think back for a moment. This could be an experience that is ongoing in your life or one that began 15 years ago. Think of a time where you successfully made a change in your life. It could be anything from quitting smoking or drinking to completing a physiotherapy program or going back to school to get a degree. What matters is that it was or is CHALLENGING to you, physically, mentally, logistically or all three.**
   1. ***Now, I’d like to hear from you about what really motivated you to BEGIN making the change…*** *(i.e.: diagnosis of a health problem, wanting to improve relationship(s), seeing someone else suffer negative consequences from a behavior, seeing someone else benefit from the change in question, being encouraged or nagged by family/friends, etc.)*
      1. ***About how much time passed between when you knew you needed or wanted to make the change and when you actually began?***
      2. ***What caused those delays?***
   2. ***So there are definitely a lot of reasons to introduce changes in our lives. But how about following-through? I’d like you to think back now and try to remember a specific hour or day where you felt especially challenged and even tempted to give up on the change.*** ***What was it that kept you going?*** *(i.e.: thought about my children not having a mother/father; thought about how disappointed I would be in myself later; called my sponsor/counselor/friend, etc.)*
   3. ***What personality traits or qualities of yours do you think helped you***? (*ie: “stubbornness”, fast learner, grit, determination, noticed progress, etc*.)
   4. ***What kind of outside factors do you think helped you*?** (*ie: encouragement from a loved one, check-in with doctor, boss, or peer; fear of adverse consequences if I failed, etc.)*
   5. ***And now, I’d like you to think of a time when you did falter or lose your way temporarily. How did you feel? And how did you get back on track?***
   6. ***Thinking back, what kind of support or resources do you think would have made it easier for you to begin the change? To follow-through?***
   7. ***What do you know now about the process that you wish you had known then? What would you tell your younger/earlier self?***

# Closing (2-5 minutes)

And now, as we conclude, is there anything final that anyone would like to add?

I’d just like to thank all of you for taking the time to share your thoughts with us and I look forward to talking with you all again at next month’s meeting.
