# Supplementary material for: The Use of Evaluation Panels During the Development of a Digital Intervention for Veterans Based on Cognitive Behavioral Therapy for Insomnia: Qualitative Evaluation Study
Source: JMIR Form Res. 2023 Mar 6;7:e40104. doi: 10.2196/40104 (PMC10028512; doi:10.2196/40104)
Supplement: Multimedia Appendix 2 [file formative_v7i1e40104_app2.docx]

U.S. Department of Veterans Affairs –

Mental Health Services

## SPOUSEs PANELS – Round 1

## Moderator Guide

## MARCH 2017

Research OBJECTIVES:

1. Gather feedback from spouses/partners on their ability and willingness to influence their veteran to seek outside help or resources.
2. gather feedback on how spouses/partners find resources and determine which ones they recommend to their veteran partners and why.
3. gather feedback from spouses/partners about their role in helping their veteran partners make changes in their lives.

#### SCHEDULE

| Date | Time (EST) |
| --- | --- |
| Tuesday, March 21, 2017 | 6:00 – 7:00 pm EST |
| Wednesday, March 22, 2017 | 3:00 – 4:00 pm EST |

# INTRODUCTION TO PANEL (10 minutes)

Thank you for joining us today. I’m Elizabeth Downs and I’ll be moderating today’s discussion.

Today’s discussion is designed to get feedback from Spouses and Partners of Veterans, such as yourself, in order to help the Department of Veterans Affairs (which we will refer to as VA) better understand life and health issues relating to your Spouse/Partner and your family in order to improve the technologies and services that VA provides.

Apart from some general questions, we’ll talk about your thoughts on online “self help” programs. We’ll also talk about your role in helping your Veteran Spouse or Partner find and get information and help relating to health and personal issues as well as ways that all of you have supported your Spouses/Partners when they have tried to make changes in their lives. I’m really looking forward to hearing all of your opinions and feedback!

As we move through the discussion today, I’d like you to be open and honest. There are no right or wrong answers. It’s okay to disagree with one another; I want to hear a range of opinions, so please do speak freely. Please just remember to respect the opinions of others in the group.

Please remember that any information collected will be kept private, no one outside of this meeting will know who said what – your personal identities will not be shared with VA. This session is being recorded, but for our analysis only.

We have a lot of ground to cover, so I may jump around at times or not call on everyone for each question. If you have something to add please just chime in or use the chat feature. We want this to be a conversation!

My colleague, Janis, who you spoke to for your recent technical check, is going to do a quick review of a few technical features before we begin. We will also do a roll call and hear a little bit about each of you.

With that, let’s get started.

**Technical Review by Janis.**

**Back to Elizabeth for Roll Call:** To kick things off, I’d like each of you to provide a quick introduction: give us your first name, which branch of the Military your Spouse or Partner served in, where you live now, and something significant going on in your life right now. Nothing too personal, just a little about yourself.

(Moderator – *Elizabeth will briefly reintroduce herself in this format to give respondents an idea of what they should say. Then, Elizabeth to call out respondents one by one, by first name. Prompt each individual attendee to provide their introduction.*)

Thank you all for those introductions.

OK, I think we’re ready to get started.

# PART 1: spouses’ ability and willingness to INFLUENCE THEIR VETERAN PARTNER TO SEEK OUTSIDE HELP OR RESOURCES (25 minutes)

****Content in parentheses is not to be spoken aloud, but rather used as a guide to rephrase questions if they are not understood by participants****

***So to start, I’d like for us to talk a bit about online courses and online “self help” resources …***

1. **With all of the online resources available to us these days, I’d like to understand what types of online resources you may have used or be familiar with for learning, growth or just general “self-help”. What are some of the general topics you’ve explored with online programs?** (ie: *Online college or extension courses; online fitness or nutrition programs; online stress relief/meditation; mental health/therapy programs, etc.*)
2. *****Use the Adobe Connect polling feature*** I’d like to take a quick poll here. When it comes to your spouse or partner seeking out online learning or “self help” programs (like those we’ve just talked about) which statement do you feel best matches his/her personality:**
   1. **When I need help with something, I find the resources I need and just do it.**
   2. **When I need help with something, I find the resource, and I bookmark it to look into…one day.**
   3. **Sometimes, I need help with something, but with one thing and another, I never seem to have time to find the resources, let alone commit to an online class.**
   4. **I don’t need help. But my spouse/partner/friend keeps pestering me, so I’ll just do it.**
   5. **Help? Who needs help?**
3. **When it comes to your spouse or partner needing outside help or resources, especially relating to health or personal issues, does he/she let you know directly that he/she wants your help?**
   1. ***If so, can you walk me through a typical conversation like that? How does the topic come up?***
4. **If your spouse/partner doesn’t directly ask for your help, do you notice something’s up and bring it up yourself?**
   1. ***What would usually motivate you to bring up these topics vs. waiting to see if things improve on their own?*** *(ie: “final straw” moment, big argument, keep noticing short temper, tossing and turning at night, straining family or professional relationships, etc.)*
   2. ***What would a conversation like that typically sound like? How do you begin?***
5. **Apart from speaking with you, who does your Spouse/Partner go to for help or advice, especially relating to health or personal issues?** *(ie: other Veterans, doctor, VSO, friends, parent, etc.)*
   1. ***Have you or would you ask a third person (one of your Spouse’s friends or family members) to speak with him/her or intervene in some way?***
6. *****Use the Adobe Connect polling feature. *** Let’s do another quick poll. Whether your spouse or partner asks for your advice or you bring it up yourself:**

**From your experience with your Spouse or Partner, which of these approaches would you be most likely to use when giving him/her advice (especially relating to their health or personal issues):**

- 1. **I would encourage him/her to speak to a doctor or counselor.**
  2. **I would encourage him/her to research possible resources online.**
  3. **I would research possible resources online myself and then give my spouse/partner ones that look good to me.**
  4. **I would talk to friends or family who I think have dealt with the same kinds of issues to see what they would recommend.**
  5. **I would just listen and talk through it with my spouse/partner. I wouldn’t recommend any outside resources.**

1. **For those of you who would research possible resources online yourselves or ask friends/family, how would you feel about recommending an online course or “self-help” program to your spouse/partner?**
   1. **What features would the program need to have for you to recommend it to your spouse/partner?** *(i.e.: anonymous/no registration required; have support or accountability built in via phone or e-mail; be free; be specifically made for Veterans; be provided by VA, etc.)*
   2. **Would you go through the program or course yourself before recommending it? Why or why not?**
   3. **Would you follow-up with your spouse/partner after recommending an online self-help program of some sort? Why or why not?**
2. ***If applicable:* For those who wouldn’t recommend any outside resources, why wouldn’t you?** (*ie: I know my spouse wouldn’t want that; I don’t feel comfortable recommending things I don’t personally understand/know more about; It’s not my role as a spouse/partner, etc.)*
3. *****Use the Adobe Connect polling feature*** One more quick poll... If you had to choose one phrase that you think would sum up your Spouse’s/Partner’s attitude when beginning a new online learning or “self-help” program (like any of the ones we talked about earlier), which of these would it be?**
   1. **“I’ve got this. Bring it on.”**
   2. **“I think I can do this. I’ll give it a try.”**
   3. **“I’m not sure I can do this, but it’s worth a shot.”**
   4. **“There’s no way this is going to work, but I’ll try.”**
   5. **“What a waste of time. Here goes nothing.”**

# PART 2: decision-making and follow-through (20 minutes)

***Thank you so much for all of that great feedback. Now we’re going to talk a bit more broadly about challenging life changes that your Spouses/Partners have made and your role in the process...***

1. **I’d like you to think back for a moment. This could be an experience that is ongoing or one that began 15 years ago. Think of a time during your relationship where your Partner/Spouse successfully made a change in his/her life. It could be anything from quitting smoking or drinking to losing weight, completing a physiotherapy program or going back to school to get a degree. What matters is that it was or is CHALLENGING : physically, mentally, logistically or all three.**
   1. ***Now, I’d like to hear from you about what you feel that your role was or is in that process…*** (*ie: encouraged spouse to begin or continue; gave an ultimatum; general emotional support; logistical support such as taking on more responsibility for the family/work, etc.)*
   2. ***If you can remember, About how much time passed between when your Spouse/Partner knew he/she wanted to make the change and when he/she actually began?***
      1. ***What caused delays?***
      2. ***Do you feel that you were able to affect your Spouse’s/Partner’s decision to begin?***
   3. ***And how about follow-through? I’d like you to think back now and try to remember a specific hour or day where you saw that your Spouse/Partner was having a hard time and may have even been tempted to give up. What do you think kept him/her going?*** ***What was your role in that?***
   4. ***And now, I’d like you to think of a time when your Spouse/Partner did falter or lose his/her way temporarily. How did he/she get back on track? What was your role in that?***
   5. ***Finally, thinking back, what kind of support or resources do you think would have made it easier for you to support your Spouse/Partner in the beginning of the process? To follow-through?***

# Closing (2-5 minutes)

And now, as we conclude, is there anything final that anyone would like to add?

I’d just like to thank all of you for taking the time to share your thoughts with us and I look forward to talking with you all again at next month’s meeting.
