# Supplementary material for: The Use of Evaluation Panels During the Development of a Digital Intervention for Veterans Based on Cognitive Behavioral Therapy for Insomnia: Qualitative Evaluation Study
Source: JMIR Form Res. 2023 Mar 6;7:e40104. doi: 10.2196/40104 (PMC10028512; doi:10.2196/40104)
Supplement: Multimedia Appendix 3 [file formative_v7i1e40104_app3.docx]

**Moderator Guide - Veterans**

**Path to Sleep**

**Round 2**

**FINAL April 7, 2017**

**OBJECTIVE**

To walk participants through one of the modules (Sleep Diary) and one interactivity (Sleep Prescription Calculator) so they can evaluate for:

- Content
- Design
- Overall Usability (ease)

**INTRODUCTION**

Thank you for joining us today. As you know, I am **[**Facilitator]and I’ll be your moderator today.

These panels are designed to capture feedback from Veterans in order to help VA design, develop, and deliver SELF-HELP online tools for Veterans.

Today we will be looking at some MOCK UPS (or BETA SITES) for an online tool. As before, I may jump around or not call on everyone for each question. If you have something to add please chime in or use chat feature. We want this to be a conversation! And as always, I want you to be to be open and honest. There are no wrong answers. Especially since we are asking for your opinions and ideas on websites and tools to make them better for Veterans.

Any Information collected will be kept private – your personal identities will not be shared with the VA. Additionally, this session is being recorded to capture information, for our evaluation purposes only.

So to get warmed up, I’m going to start with an easy and fun (I hope) question. What was the LAST MOVIE you saw, in the theater? I’ll start. I saw Hell or High Water (Jeff Bridges movie) last summer.

*Call on each Participant.*

Thank you for that! And I think we are ready to dig in here.

**OVERVIEW**

So, as I’m sure you remember, last time we spoke, we talked about self improvement and self help tools. Your feedback and comments were really helpful.

Right now, along those lines, VA is developing an online course to help people who are having trouble sleeping.

I’d like to show you what we have, and get your feedback and thoughts.

**Starting the Conversation**

**[**Facilitator]: To start with, I want to talk a little about insomnia and difficulty sleeping.

Have you ever had trouble sleeping, even for a few days? I’d like you to type into the Chat Box just a few words that describe how you felt during the day, as a result of the sleeping issues.

*Acknowledge comments/ chats*

**[**Facilitator]: According to physicians, [insomnia](http://sleepdisorders.sleepfoundation.org/chapter-2-insomnia/what-is-insomnia/) is difficulty falling asleep or staying asleep, even when a person has the chance to do so. People with insomnia usually experience one or more of the following [symptoms](https://sleepfoundation.org/insomnia/content/symptoms): fatigue, low energy, difficulty concentrating, mood disturbances, and decreased performance in work or at school.

Now imagine those systems stretching out for three months or more! You can imagine how that would impact your life, your relationships, even your work, and my lead you to seek out help. That is the definition of insomnia:

**Chronic insomnia**is disrupted sleep that occurs at least three nights per week and lasts at least three months. *(Show definition onscreen)*

The online course that VA is developing is really for people who have true insomnia. That is because insomnia is frequently the cause of a number of other issues, including depression and anxiety.

As you view the slides and websites that I show, keep that in mind.

The other thing I want to say is – we want your help on improving these websites that you are going to see. You all surf the web and use sites and online apps and tools, so you know what works for you and what you like. It is those insights and opinions that we want so don’t be shy about sharing.

**GO: CHAPTER 4 SLIDE 1**


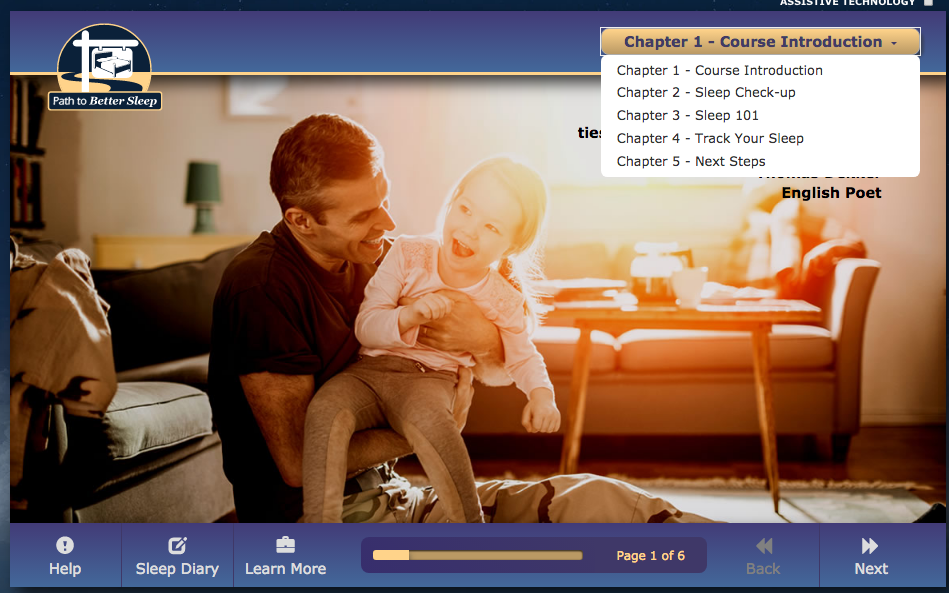


**[**Facilitator]*to show the Course Chapter 4*

*Show Drop down list*

Last time we spoke, you all talked about how important TRACKING IS TO YOUR PROCESS during some type of self help or self improvement effort.

Here we are on VA’s Sleep Course site – Path to a Better Sleep – and this section is all about tracking your sleep.

**[**Facilitator]*to show Chapter 4, slide 1 - 2*

We are going to talk about the Sleep Diary that this website offers.

**GO: CHAPTER 4 SLIDE 3**


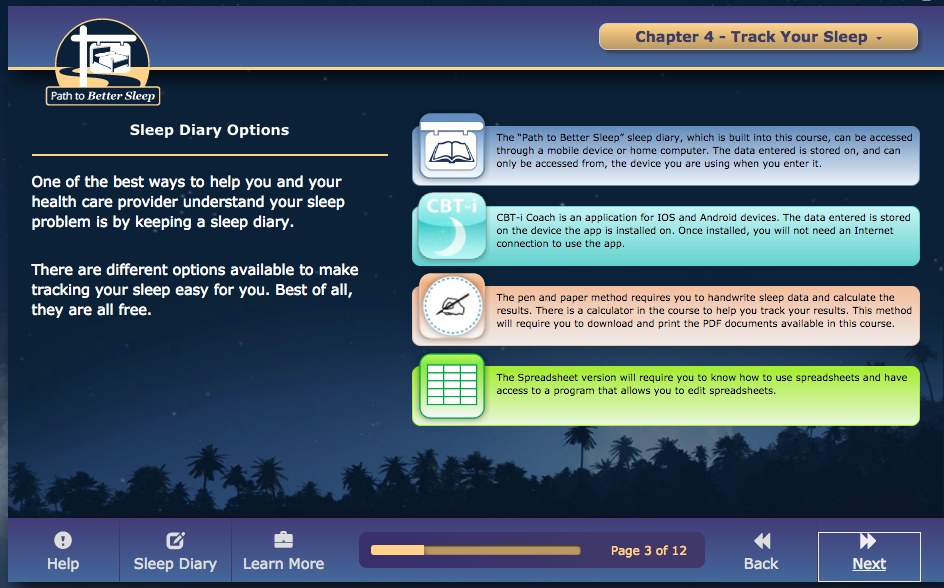


1. Here is the webpage introducing the Sleep Diary. I will read the text, and then I want to pause for a few seconds to let you take a look at it and then I will ask you for first impressions ([Facilitator] *reads the text on the left, pause for 7 seconds).*
2. What are the first things that you notice about this page?
3. What do you understand from this page? Is there an action you are expected to take?

(Do you think it is easily understood?)

1. Based on this text and the information on this page, what would you do next?
2. POLL QUESTION:

Based on this page alone, and the information on it, which one of these choices would you use?

- Path to Better Sleep sleep diary
- CBT-I Coach
- Pen and Paper Method
- Spreadsheet Version
- None of these

[Facilitator]: Interesting answers. Now we are going to move to the next page in the Sleep Diary section.

**GO: CHAPTER 4 SLIDE 4**


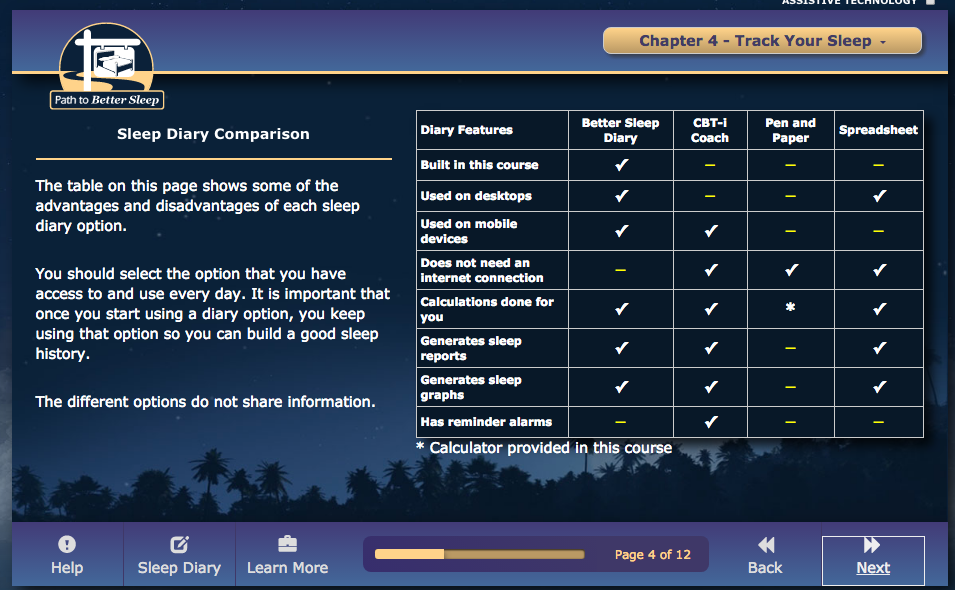


I will again read the text.

[Facilitator] *reads the text on the left and walks through each method and categories.*

The table explains specific features of each Sleep Diary method from the previous page we just looked at.

1. First, let’s talk about the table itself:
2. What do you think it means “Built in This Course”?
3. What do you think it means “Has reminder Alarms”?
4. Do you think the chart is easy to read? If not, why not?
5. Now let’s talk about the specific features of each Diary.
6. Which of these features would be most useful or important to you? Why?
7. What do you think of the categories shown? Is there a category/information you wish it had? ([Facilitator] *this could be amount of time it takes to set up, programs needed to run it on desktop, etc*)
8. Type into the chat box which type of sleep diary you would choose now, that we’ve seen the chart on this page.
9. Based on the information on this page, would you change your initial poll response? *SHOW PREVIOUS POLL RESPONSES*
   1. Why?

**GO: CHAPTER 4 SLIDE 5**


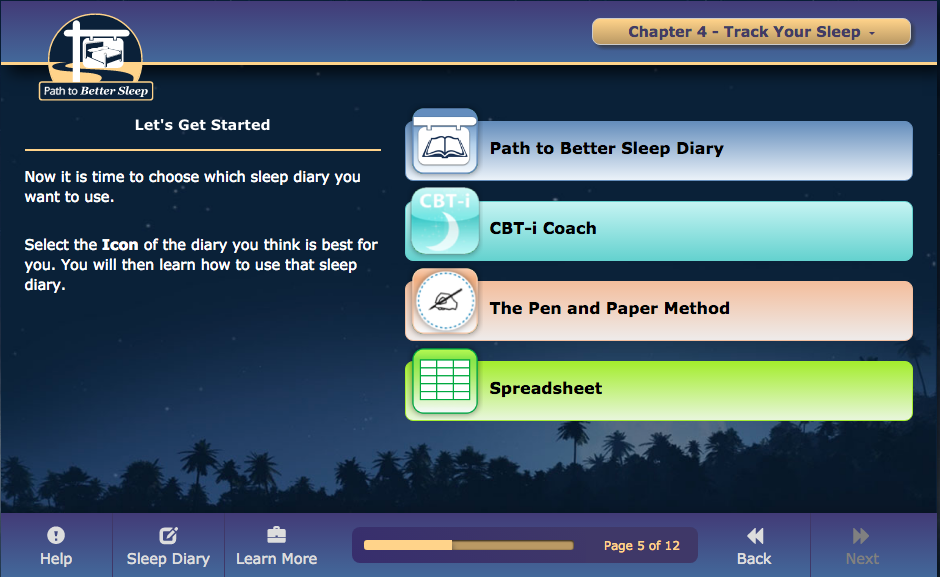


Ok, we are moving on to the next page. Again I will read the text.

[Facilitator] *reads the content on the left and each item on right*

1. In looking at this page what do you think of the layout?
2. What do you think about the information provided? (PROBE:Is it clear that there are 4 different ways to keep a Sleep Diary or track your sleep?)
3. What do you think of the names of the 4 methods? What do you think they mean/are?
   - If not, how would you call them?

So for this exercise, we are going to select the first one: Path to Better Sleep Sleep Diary

**GO: CHAPTER 4 SLIDE 6**


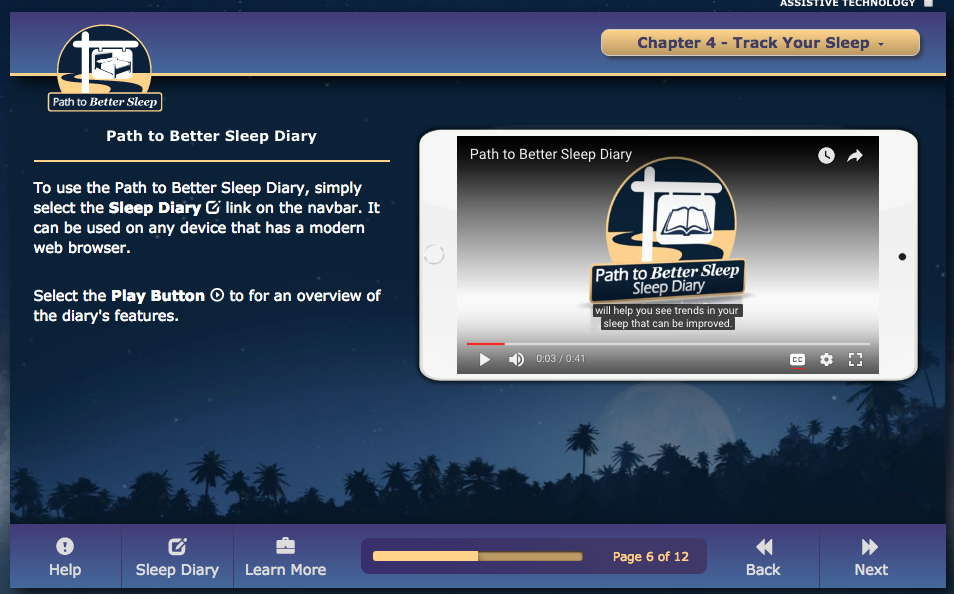


[Facilitator] *reads text*

1. On this page, what do you think you are being asked to do?
2. What would you click?
3. Is it clear/obvious? If not, why not? What should it say?

**(Go to Sleep Diary Slide)**

So now we have clicked the Sleep Diary button. And here we are.


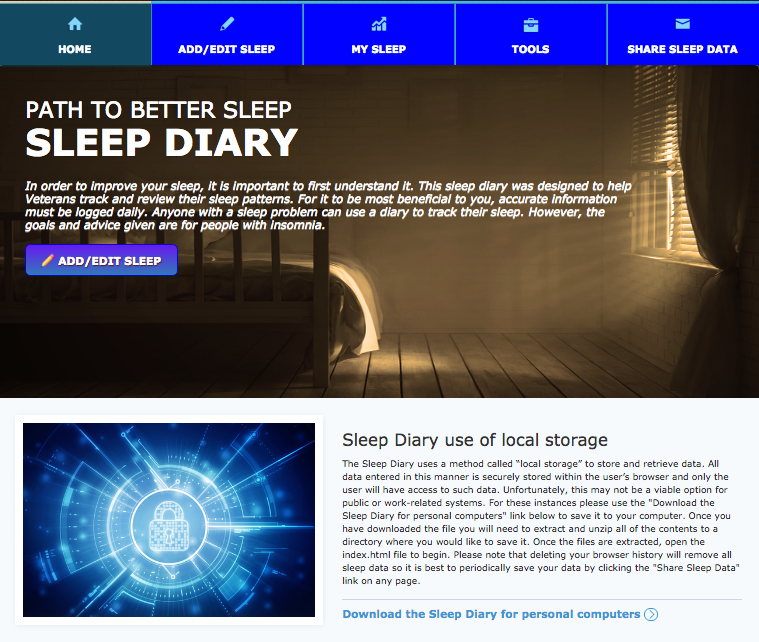


1. Here we are at the Sleep Diary page. *[Facilitator] reads content in primary box.* Again, I want to give you a few seconds to look at it and then we will discuss (*pause 10 seconds*)
2. What is your opinion on the language? (PROBE – Is it clear and understandable?)
3. What do you think this is for?
4. What would your next step be?
5. Now look at the box in the light grey *([FACILITATOR] mouse over).* We know we need to rewrite this, so I’m asking you to take some time to read it over. Then in the chat box, write 1 -3 sentences summarizing the content. *(20 seconds pause)*

Thank you! Now we are going on the Sleep Diary.

**GO: SLEEP DIARY**


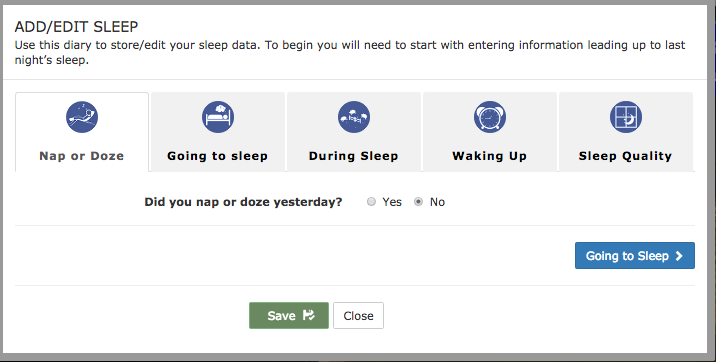


1. Sleep Diary

So to put this in context again, just like many of you told us about your experiences tracking your nutrition or fitness progress and how that helped keep you focused and moving forward, this Sleep Diary is meant to help folks who have insomnia to keep track of their sleep. It helps them find issues that might be keeping them from sleeping well - like taking naps or the time of night they go to sleep - as well as find the patterns in things that they do or don't do on nights they DO sleep well.

So I’m going to show you the process of the Sleep Diary.

[Facilitator] *will show the 5 steps, walking through the set up and flow – clicking on each step.*

1. In looking at the set up of the Sleep Diary page, what do you think of the flow of this page?
2. What are your thoughts on the names/titles for each section (Nap or Doze, Going to Sleep, etc.)?
3. What would you like to see added or clarified?
4. What would you do next?

Now that you have looked at the Sleep Diary, designed for people with real insomnia, think about this: you said in our previous discussion that you like to track progress. Think of some of the apps you use, or online tools you may use to track your progress, whatever it may be: Fitness, Nutrition, Weight Loss.

1. Think about how you use those Tracking Apps. Can you share with me what specific feature you like about it?
2. Before we move to another section of the website, is there anything you want to add about the Sleep Diary set up?
3. Now that you have seen the Sleep Diary, is this something that you think someone who has insomnia would find useful? If not why not?
4. How would you describe it to a friend or family member?
5. POLL QUESTION: If you knew someone who was having difficulty sleeping, would you recommend this course and Sleep Diary to him/her?

Yes

No

Maybe

I don’t know

Thank you for all that great feedback! We are almost done, just a few more questions for you.

**NEXT SECTION**

Interactivity

<http://www.sleepbettercourse.com/to2/resources/interactivities/calculator/index.html>

Before I put the next website on the screen, what I want from you, as soon as you see it, is to give me your gut, knee-jerk, instant reaction – you can just blurt it out if you want. Or type into the Chat box. But I don’t want you to think about it – just react.

[Facilitator] *pulls up Calculator*

**GO: SLEEP CALCULATOR INTERACTIVITY**


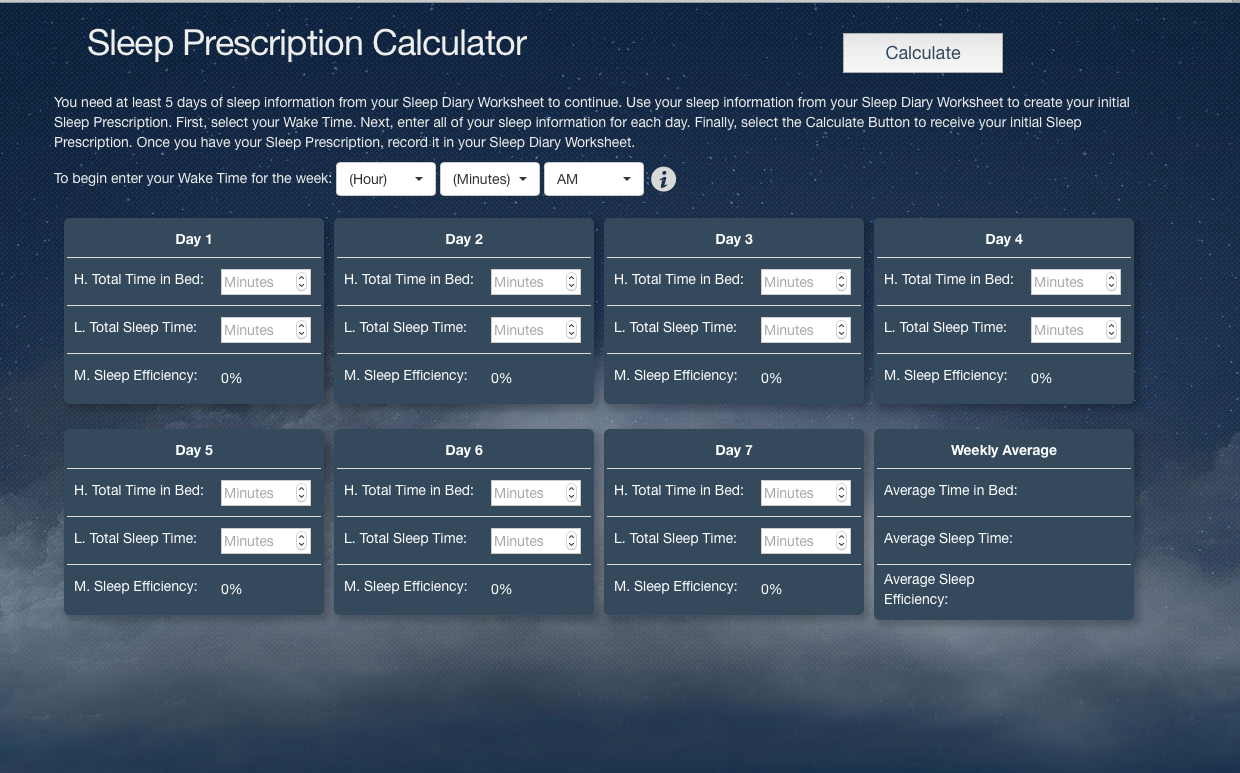


[Facilitator] *responds to reactions – PROBE* *based on reactions – Why did you say . .. . and What do you mean by?*

This is the Sleep Prescription Calculator, a tool available on the Sleep Course site.

1. As we look at this page I have some questions for you:
2. What do you think this page is for?
3. What do you think you are supposed to do on this page?
4. Let’s talk about the language – what do you think about the instructions? (*PROBE: Is the language plain and easy to understand?)*
5. What do you think of the flow or lay out?
6. Other thoughts?

**IF THERE IS TIME**

<http://www.sleepbettercourse.com/to2/index.html#02_09_007>

**GO: Action Plan**


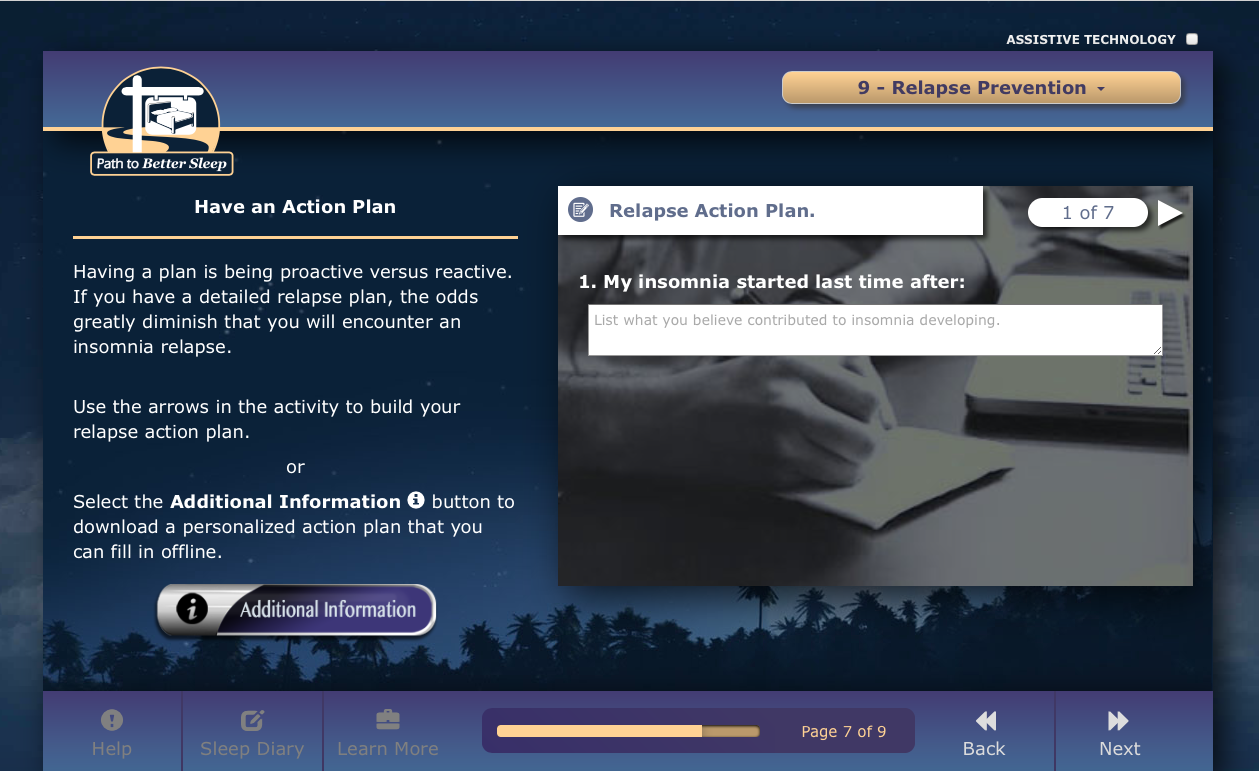


*[Facilitator] reads aloud the copy to the left*

1. In reading the text on this page, what do you think they are saying?
   1. What do you think of when you hear “Relapse Action Plan?”
   2. If you were using this, what would you do next?

*Mouse over the Relapse Action Plan – My insomnia started last time after*

1. What do you think they are asking?

*[Facilitator] click to slide 5*


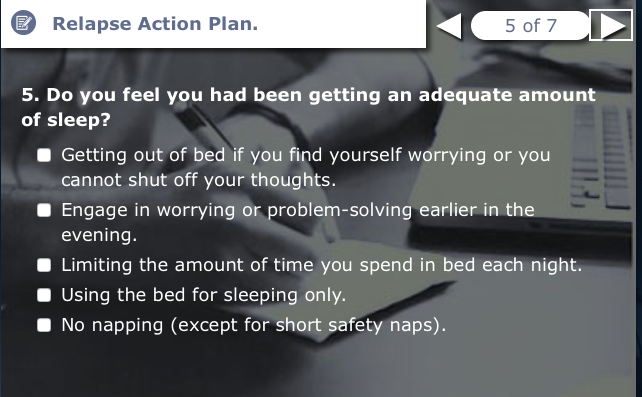


*[Facilitator] Read aloud question*

1. Without looking at the answers, what do you think the answer should be to this question? (QUESTION IS YES/NO, ANSWERS ARE NOT)
2. In looking at the answers, do they help clarify or confuse or neither?

While we won’t go through all of the questions (there are only 8), I do want you to see the end result that the program produces, based on the answers someone inputs.

*[Facilitator] show report*


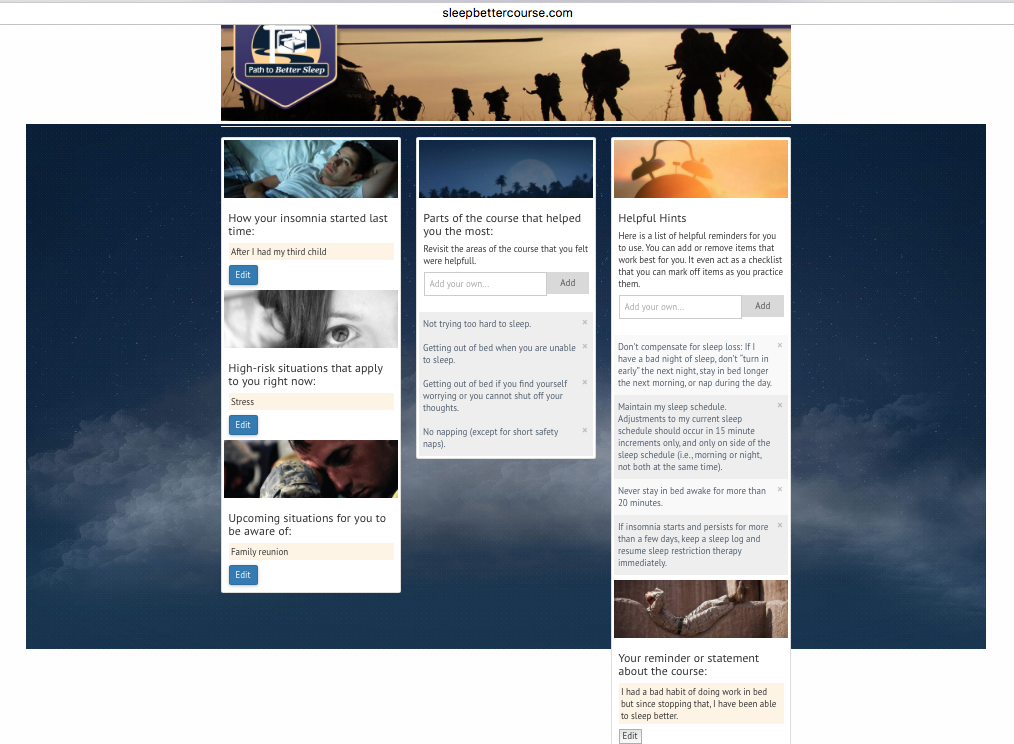


This is what they call your Relapse Prevention Plan.

1. Looking at the layout what are your first thoughts? (are the colors of the text easy to see? Do the pictures make it conducive to reading and understanding?)
2. What information is missing?
3. How useful do you think this Plan is?

**CLOSING**

So that is it for today!

I’d just like to thank all of you for taking the time to share your thoughts with me and I look forward to talking with you all again at next month’s meeting.

You will be contacted prior to that call just as we did with this one.

If you have any questions or want to reach out to us, you should all have Janis’s contact information. Thank you again and have a good evening.
