# Supplementary material for: The Use of Evaluation Panels During the Development of a Digital Intervention for Veterans Based on Cognitive Behavioral Therapy for Insomnia: Qualitative Evaluation Study
Source: JMIR Form Res. 2023 Mar 6;7:e40104. doi: 10.2196/40104 (PMC10028512; doi:10.2196/40104)
Supplement: Multimedia Appendix 4 [file formative_v7i1e40104_app4.docx]

**Moderator Guide - Spouses**

**Path to Sleep**

**DRAFT April 12, 2017**

**ROUND 2**

**OBJECTIVE**

To walk participants through one of the modules (Sleep Diary) and one interactivity (Sleep Prescription Calculator) so they can evaluate for:

- Content
- Design
- Overall Usability (ease)

**INTRODUCTION**

Thank you for joining us today. As you know, I am ELIZABETH DOWNS and I’ll be your moderator today.

These focus groups are designed to capture feedback from Veterans and Spouses of Veterans in order to help VA design, develop, and deliver SELF-HELP online tools and programs.

Today we will be reviewing some websites and online tools that are in development at VA. As before, I may jump around or not call on everyone for each question. If you have something to add please chime in or use chat feature. We want this to be a conversation! And as always, I want you to be to be open and honest. There are no wrong answers. Especially since we are asking for your opinions and ideas on websites and tools so that we can improve them.

Any Information collected will be kept private – your personal identities will not be shared with VA. Additionally, this session is being recorded to capture information, for our evaluation purposes only.

So to get warmed up, I’m going to start with an easy and fun (I hope) question. What was the LAST MOVIE you saw, in the theater? I’ll start..

*Call on each Participant.*

Thank you for that! And I think we are ready to dig in here.

**OVERVIEW**

So, as I’m sure you remember, last time we spoke, we talked about self improvement and self help tools, specifically ones you have used and those your Veteran has used.

Right now, along those lines, VA is developing an online course to help people who are having trouble sleeping.

I’d like to show you what they have so far, and get your feedback and thoughts on improving it.

**Starting the Conversation**

ELIZABETH: To start with, I want to talk a little about insomnia and difficulty sleeping.

Have you ever had trouble sleeping, even for a few days? I’d like you to type into the Chat Box just a few words that describe how you felt during the day, as a result of the sleeping issues.

*Acknowledge comments/ chats*

ELIZABETH: According to physicians, [insomnia](http://sleepdisorders.sleepfoundation.org/chapter-2-insomnia/what-is-insomnia/) is difficulty falling asleep or staying asleep, even when a person has the chance to do so. People with insomnia usually experience one or more of the following [symptoms](https://sleepfoundation.org/insomnia/content/symptoms): fatigue, low energy, difficulty concentrating, mood disturbances, and decreased performance in work or at school.

Now imagine those systems stretching out for three months or more! You can imagine how that would impact your life, your relationships, even your work, and my lead you to seek out help. That is the definition of insomnia:

**Chronic insomnia**is disrupted sleep that occurs at least three nights per week and lasts at least three months. *(Show definition onscreen)*

The online course that VA is developing is really for people who have true insomnia. That is because insomnia is frequently the cause of a number of other issues, including depression.

As you view the websites that I show, keep that in mind.

The other thing I want to say is – we want your help on improving these websites that you are going to see. You all surf the web and use sites and online apps and tools, so you know what works for you and what you like. It is those insights and opinions that we want so don’t be shy about sharing.

So I’m going to share my screen with you now. Give it a few seconds for the website to appear and please let me know if you don’t see the website on your computer. Also, please let me know if it is big enough, or if you need me to zoom in.

**GO: CHAPTER 4 SLIDE 1**


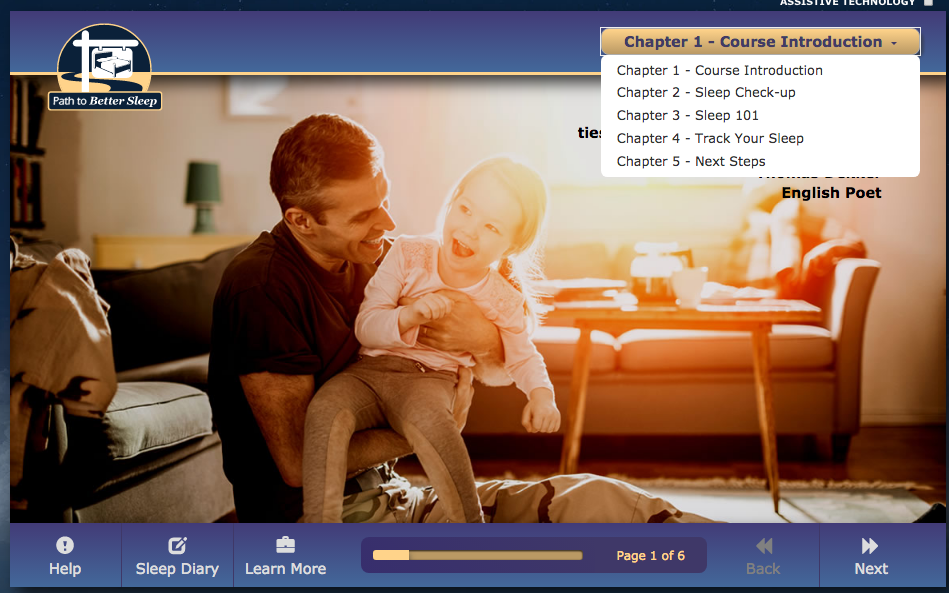


*Elizabeth to show the Course Chapter 4*

Last time we spoke, you all talked about how important TRACKING IS TO YOUR PROCESS, when you are doing some type of self help or self improvement effort, and for your Veteran as well.

Here we are on VA’s Sleep Course site – Path to a Better Sleep – and Chapter 4 of this course is all about tracking your sleep.

*Elizabeth to show Chapter 4, slide 3*

**GO: CHAPTER 4 SLIDE 3**


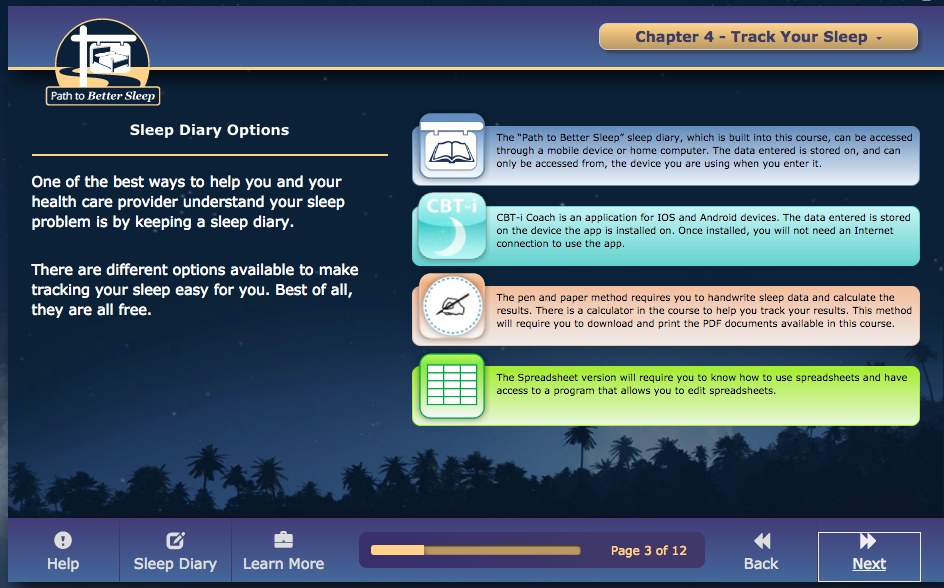


1. Here is the webpage introducing the Sleep Diary – what VA has developed for tracking. What is your first impression of this page – the lay out?
   1. What is the first thing you notice?
2. I will read the text, and then I want to ask some questions
3. What do you understand from this page? What is the action you are expected to take?

(Do you think it is easily understood? )

1. Based on this text and the information on this page, what would you do next?
2. POLL QUESTION:

Based on this page alone, and the information on it, which one of these choices would you use?

- Path to Better Sleep sleep diary
- CBT-I Coach
- Pen and Paper Method
- Spreadsheet Version
- None of these

Elizabeth: Interesting answers. Now we are going to move to the next page in the Sleep Diary section.

**GO: CHAPTER 4 SLIDE 4**


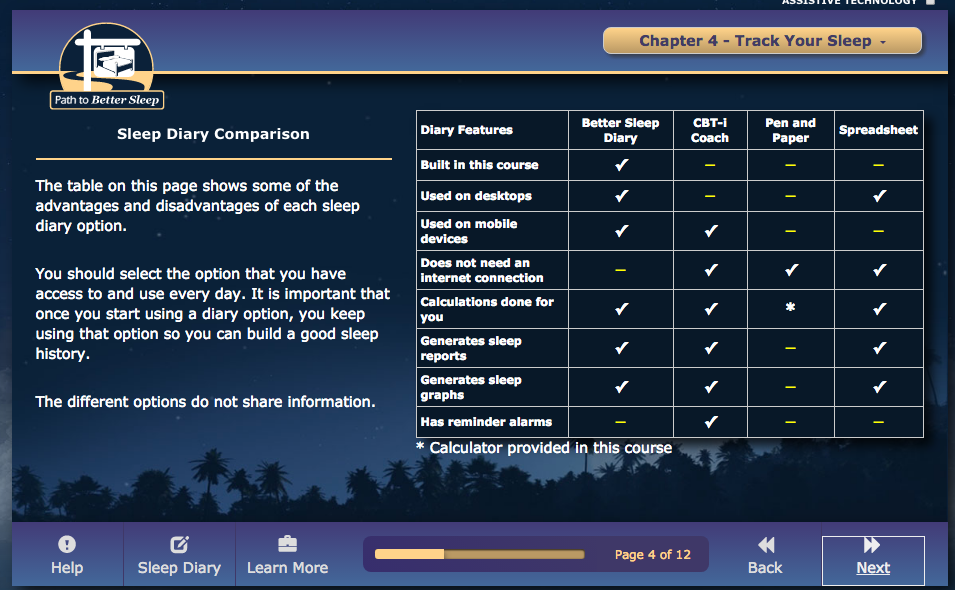


1. Again, what are your first impressions of this page and how it is laid out? <*Is it clean, is it confusing? Too much information too little?>*

I will again read the text.

*Elizabeth reads the text on the left and walks through each method and categories.*

The table explains specific features of each Sleep Diary method from the previous page we just looked at.

1. First, let’s talk about the table itself:
2. What do you think it means “Built in This Course”?
3. What do you think it means “Has reminder Alarms”?
4. Do you think the chart is easy to read? If not, why not?
5. Now let’s talk about the specific features of each Diary.
6. Which of these features would be most useful or important to you? Why?
7. What do you think of the categories shown? Is there a category/information you wish it had? (*ELIZABETH this could be amount of time it takes to set up, programs needed to run it on desktop, etc*)
8. Type into the chat box which type of sleep diary you would choose, now that we’ve seen the chart on this page.
9. Based on the information on this page, would you change your initial poll response?
   1. Why?
10. If your Veteran had insomnia, and was going through this course, which one do you think he or she would select? WHY?

**GO: CHAPTER 4 SLIDE 5**


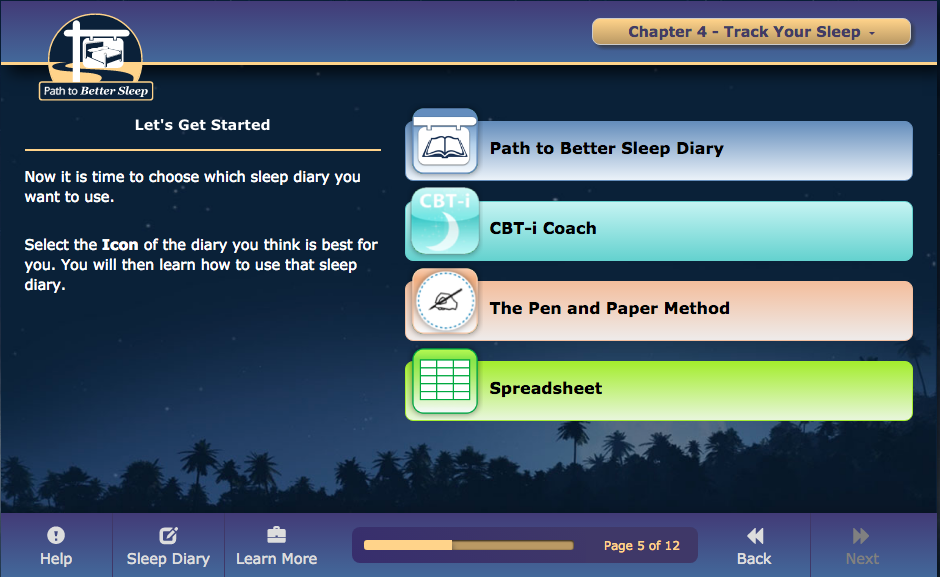


Ok, we are moving on to the next page. Again I will read the text.

*Elizabeth reads the content on the left and each item on right*

1. In looking at this page what do you think of the layout?
2. What do you think about the information provided? (PROBE:Is it clear that there are 4 different ways to keep a Sleep Diary or track your sleep?)
3. What do you think of the names of the 4 methods? What do you think they mean/are?
   - If not, how would you call them?

So for this exercise, we are going to select the first one: Path to Better Sleep Sleep Diary

**GO: CHAPTER 4 SLIDE 6**


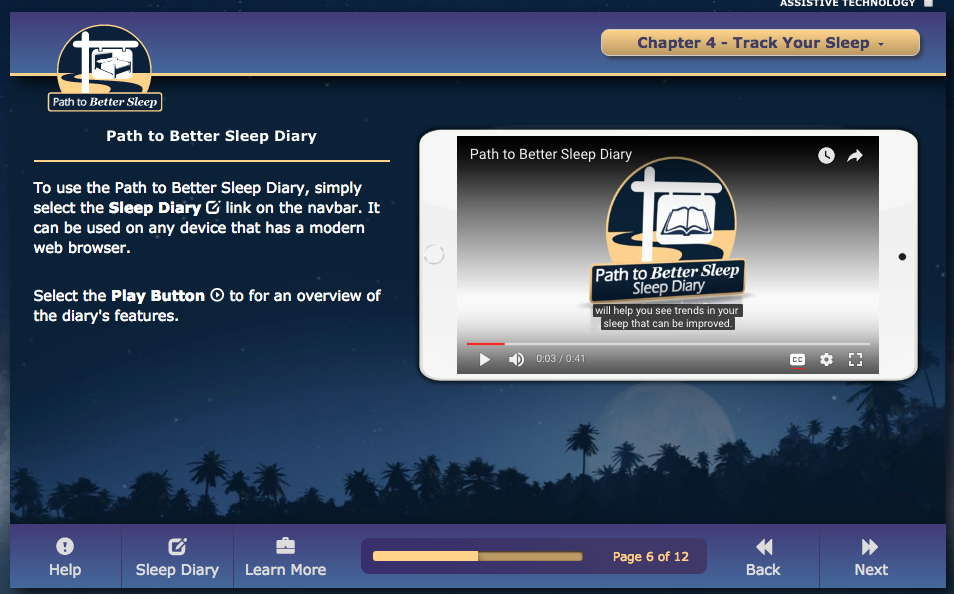


*Elizabeth reads text*

1. On this page, what do you think you are being asked to do?
2. What would you click?
3. Is it clear/obvious? If not, why not? What should it say?

**(Go to Sleep Diary Slide)**

So now we have clicked the Sleep Diary button. And here we are.


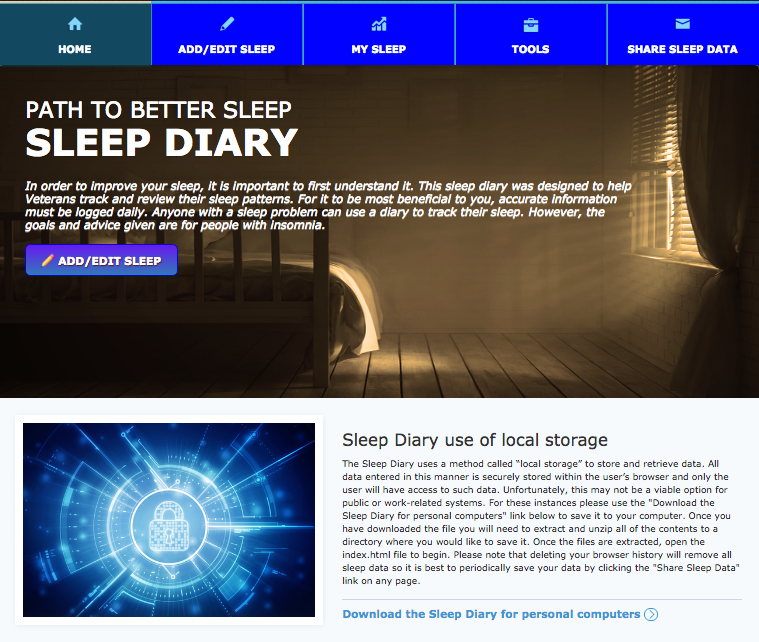


AS YOU LOOK AT THIS PAGE, WHAT ARE YOUR INITIAL THOUGHTS?

1. Ok. Let’s talk about the wording. I’m going to read the text at the top part and then we can talk about it. *Elizabeth reads content in primary box.*
2. What is your opinion on the language? (PROBE – Is it clear and understandable?)
3. What do you think this is for?
4. What would your next step be?
5. Now look at the box in the light grey *(ELIZABETH mouse over).* We know we need to rewrite this, so I will read the text to out loud. Then in the chat box, I’d like you to write 1 -3 sentences – or even just bullet points - summarizing what you think it is trying to say..

Thank you!

NOW BEFORE WE GO TO THE ACTUAL SLEEP DIARY, LET’S PUT IT IN CONTEXT.

Just like many of you told us about your experiences tracking your nutrition or fitness progress and how that helped keep you focused and moving forward, this Sleep Diary is meant to help folks who have insomnia to keep track of their sleep. It helps them find issues that might be keeping them from sleeping well - like taking naps or the time of night they go to sleep - as well as find the patterns in things that they do or don't do on nights they DO sleep well.

So I’m going to show you the process of the Sleep Diary.

**GO: SLEEP DIARY**

It has these 5 stages of tracking your sleep. We will quickly go through this, then I will ask you some questions. If you have any questions or comments while I go through it, please share!

*Elizabeth will show the FIRST PAGE AND GO THROUGH THE 5 steps, walking through the set up and flow*


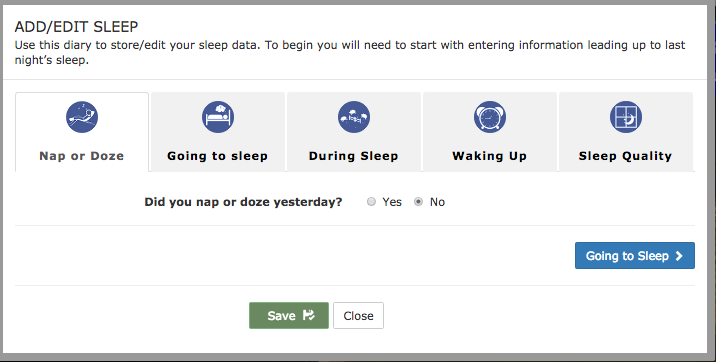


1. Sleep Diary
2. In looking at the set up of the Sleep Diary page, what do you think of the flow of this page?
3. What are your thoughts on the names/titles for each section (Nap or Doze, Going to Sleep, etc.)?
4. What would you like to see added or clarified?
5. What would you do next?

OK LET’S WALK THROUGH THE WHOLE THING NOW: (CLICK ON EACH STEP)

1. Now that you have seen the Sleep Diary, is this something that you think someone who has insomnia would find useful? If not why not?
2. How would you describe it to your Veteran if you thought they needed it?
3. POLL QUESTION: If your Veteran was having difficulty sleeping, would you recommend this course and Sleep Diary to him/her?

Yes

No

Maybe

I don’t know

Thank you for all that great feedback! We are almost done, just a few more questions for you.

**NEXT SECTION**

Interactivity

The next website I’m going to show you is an activity that Veterans with insomnia can use through this course.

Before I put the website up on the screen, what I want from you, as soon as you see it, is to give me your gut, knee-jerk, instant reaction – by typing it into the Chat box. This could be one word, one sentence, and emoji, whatever your reaction to it is..

*Elizabeth pulls up Calculator*

<http://www.sleepbettercourse.com/to2/resources/interactivities/calculator/index.html>

**GO: SLEEP CALCULATOR INTERACTIVITY**


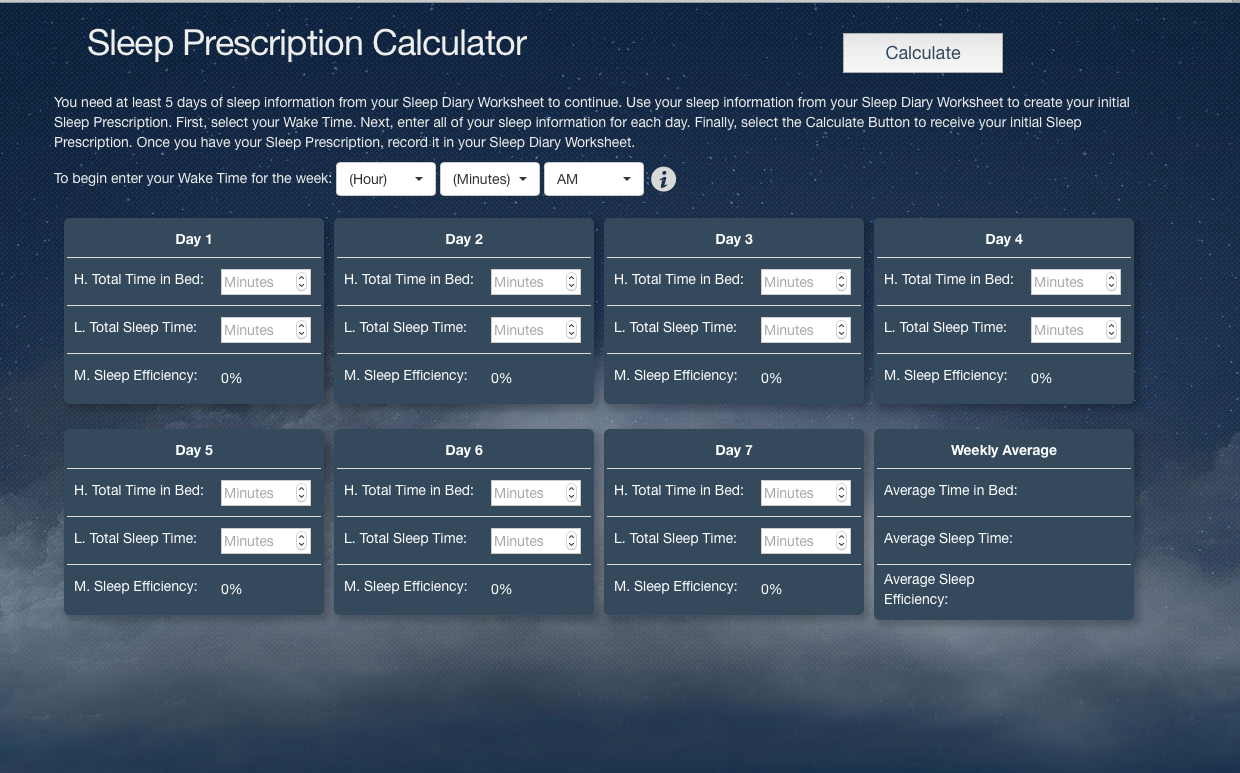


*Elizabeth responds to reactions – PROBE* *based on reactions – Why did you say . .. . and What do you mean by?*

This is the Sleep Prescription Calculator, a tool available on the Sleep Course site.

LET’S TALK ABOUT YOUR COMMENTS:

1. As we look at this page I have some questions for you:
2. What do you think this page is for?
3. What do you think you are supposed to do on this page?
4. I’m going to read the instructions at the top:
5. Let’s talk about the wording – what do you think about the instructions? (*PROBE: Is the language plain and easy to understand?)*
6. What do you think of the flow or lay out?
7. Other thoughts?

Great! Thank you for all that!

**IF THERE IS TIME**

Ok, the next site I’m going to show you is still part of the Sleep Course.

<http://www.sleepbettercourse.com/to2/index.html#02_09_007>

**GO: Action Plan**


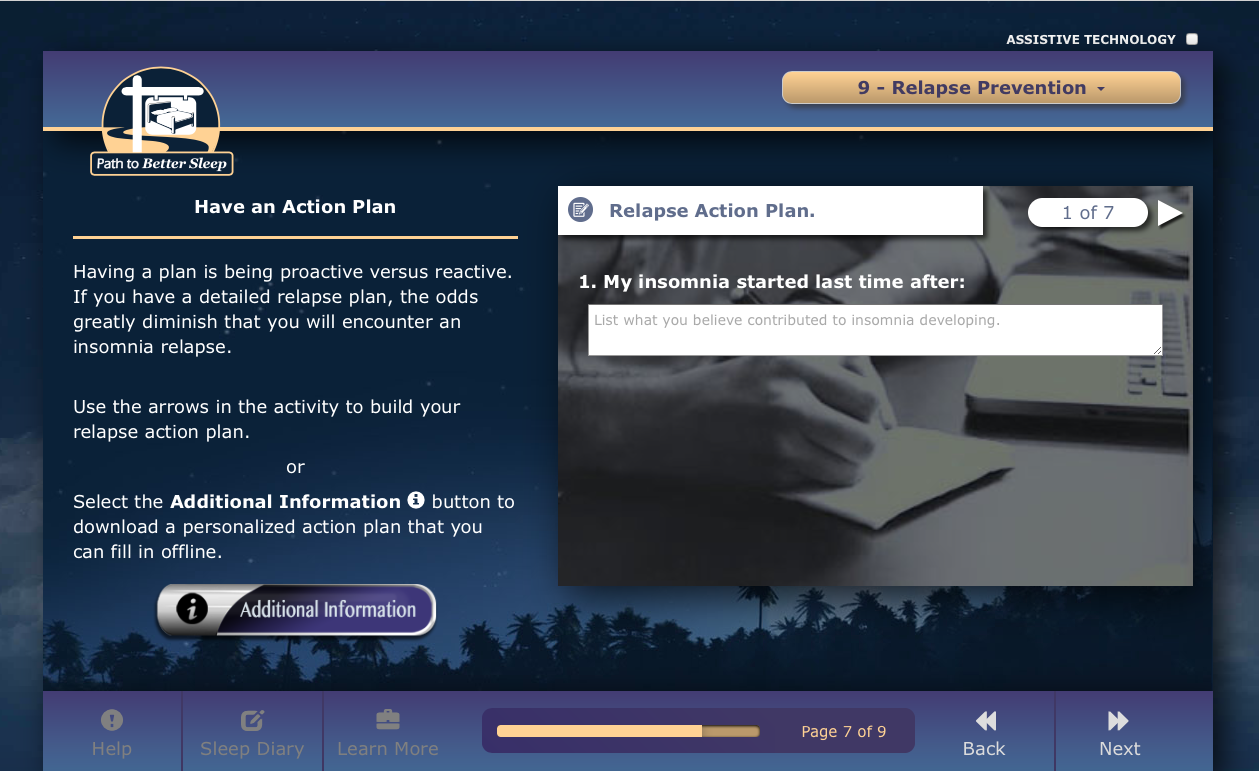


I’m going to read the section on the left and then we will talk about how it is worded.

*Elizabeth reads aloud the copy to the left*

1. In reading the text on this page, what do you think they are saying?
   1. What do you think of when you hear “**Relapse?”**
   2. If you were using this, what would you do next?

*Mouse over the Relapse Action Plan – My insomnia started last time after*

1. What do you think they are asking?

Ok we are going to go through the steps of the Action Plan and as I fill it out, I want to know your opinion of it.

<SLIDE 4 and 5 probe>


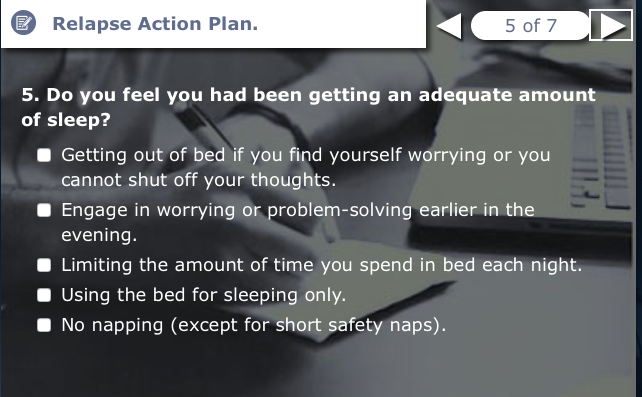


*Elizabeth Read aloud question*

1. Without looking at the answers, what do you think the answer should be to this question? (QUESTION IS YES/NO, ANSWERS ARE NOT)
2. In looking at the answers, do they help clarify or confuse or neither?

While we won’t go through all of the questions (there are only 8), I do want you to see the end result that the program produces, based on the answers someone inputs.

*Elizabeth show report*


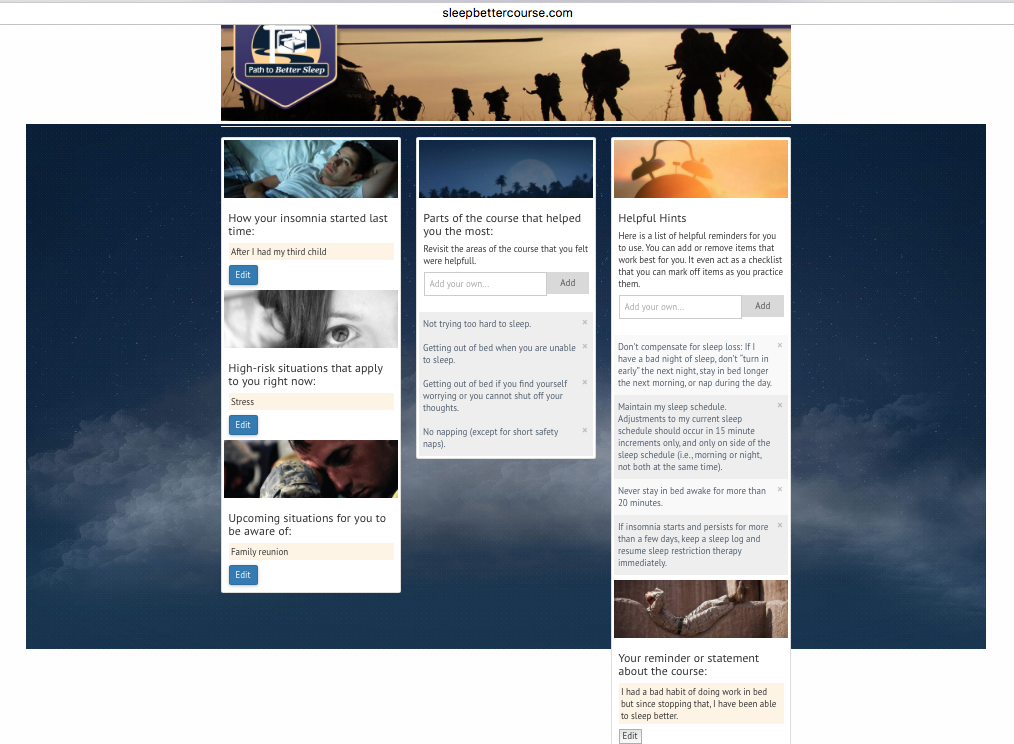


This is what they call your Relapse Prevention Plan.

1. Looking at the layout what are your first thoughts? (are the colors of the text easy to see? Do the pictures make it conducive to reading and understanding?)
2. What information is missing?
3. How useful do you think this Plan is?

**CLOSING**

So that is it for today!

I’d just like to thank all of you for taking the time to share your thoughts with me and I look forward to talking with you all again at next month’s meeting.

You will be contacted prior to that call just as we did with this one.

If you have any questions or want to reach out to us, you should all have Janis’s contact information. Thank you again and have a good evening.
