# Supplementary material for: The Use of Evaluation Panels During the Development of a Digital Intervention for Veterans Based on Cognitive Behavioral Therapy for Insomnia: Qualitative Evaluation Study
Source: JMIR Form Res. 2023 Mar 6;7:e40104. doi: 10.2196/40104 (PMC10028512; doi:10.2196/40104)
Supplement: Multimedia Appendix 6 [file formative_v7i1e40104_app6.docx]

U.S. Department of Veterans Affairs –

Mental Health Services

## SPOUSE Panels – Round 3

## Moderator Guide

## MAy 2017

Research OBJECTIVES:

1. Gather feedback from spouses on the path to better sleep fact sheet.
2. Gather feedback on the path to better sleep course landing page and course GUIDE map.
3. Gather feedback on the clarity of course material in the first part of Mile Marker 1 (sleep scheduling).

#### SCHEDULE

| Date | Time (EST) |
| --- | --- |
| May 23, 2017 | 6:00 – 7:00 pm ET |
| May 24, 2017 | 3:00 – 4:00 pm ET |

# INTRODUCTION TO PANEL (10 minutes)

**Janis to do quick tech refresh (highlight the part about playing VIDEO as we’ll watch a video) and roll call.**

**Elizabeth:**

Thank you for joining us again today for our third and final focus group. As you may remember, at our last meeting, we had a look at the Sleep Diary feature of VA’s upcoming “Path to Better Sleep” course. You all gave us some very useful feedback on that diary and today we would like to look more at the “Path to Better Sleep” course and see what you think about some other parts of it.

Just like before, I’d like you to be open and honest. There are no right or wrong answers. Please just remember to respect the opinions of others in the group.

Please remember that any information collected will be kept private, no one outside of this meeting will know who said what – as always, your personal identities will not be shared with VA. This session is being recorded, but for our analysis only.

We have a lot of ground to cover, so I may jump around at times or not call on everyone for each question. If you have something to add please just chime in or use the chat feature. Even if we don’t get to look at every comment in the chat in real-time, we do include these comments in our analysis.

Great. With that, let’s get started.

# PART 1: Review path to better sleep fact sheet, landing page & Course guide map (20 minutes)

So to begin, I’d like us to have a look at the promotional Fact Sheet that we are working up for the Path to Better Sleep course. Last month, we saw some of the sleep tracking features of the course. This month, we’ll be looking at a bit more of that course, but first I’d like your impressions of the informational fact sheet.

Pull up PDF: SLEEP COURSE FACT SHEET.


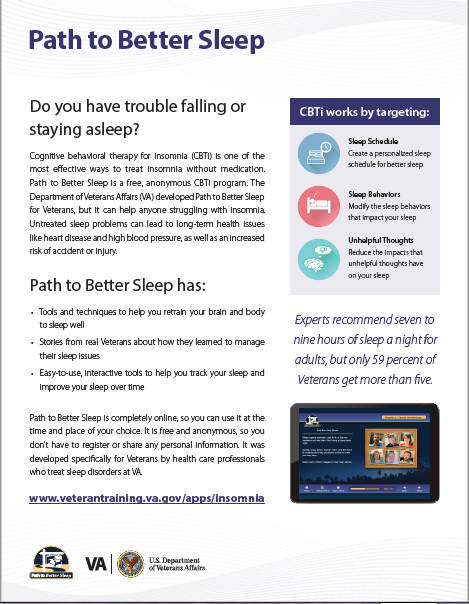


1. **Please enter into the chat box any initial impressions of the Fact Sheet - As always we would like your honest and open feedback on look & feel, content, organization and usefulness. (**Elizabeth to call on a few people to elaborate**)**
   1. ***If your Veteran has insomnia, how likely would you be to recommend the Path to Better Sleep course based on this fact sheet?***
   2. ***If you yourself have insomnia, would you look into the course after seeing this fact sheet?***


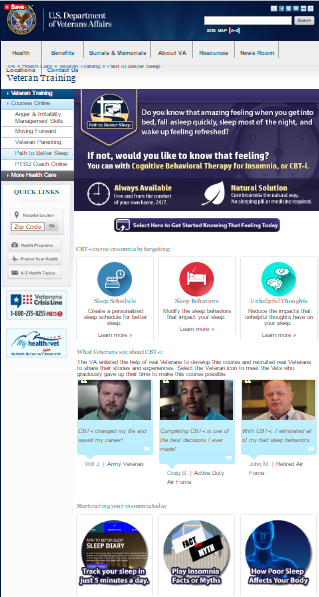


Thank you for that feedback. So at this final meeting, we’re going to go through a bit more of the Sleep course we saw parts of last time. We want to provide context and also to get your input on some of the real “meat” of the course itself. The sleep tracking we looked at last time is just one part of the larger course. The course itself is built upon the fundamentals of Cognitive Behavioral Therapy for Insomnia, or CBT-i. Some of you may remember seeing that acronym on one of the Sleep Diary options last month.

So I’m going to show you the landing page for the course and get your general feedback, and then we’re going to look at the course outline that really lays out the content of the course. Just like before, I’ll ask for your feedback on individual sections. With that, let’s get started. Pull up Sleep Course landing page:

<http://www.sleepbettercourse.com/landing2/landing3/> index_may4.html#

1. Please take a moment to skim this landing page and please type your general impressions into the chat box: These could be about the look & feel, the content, the layout or organization. Just whatever jumps out at you and then we’ll discuss (Elizabeth to call on a few people to elaborate.)
2. Seeing this landing page, if you or your Veteran had sleep issues such as the ones mentioned (not falling asleep quickly, not sleeping most of the night or not waking up feeling refreshed), where would you be drawn on this page?
   1. What are your impressions about the Veteran testimonials? Would these affect your decision to look further into the course? Why or why not?

Thank you for that feedback. We’re going to go ahead and go the Sleep Course Guide Map now and see the organization of the therapeutic part of the course.

Course Guide Map (Click on Course Guide Map menu to see the drop-down of chapters that correspond to the Mile Markers on the map). http://www.sleepbettercourse.com/to2/index.html#dashboard


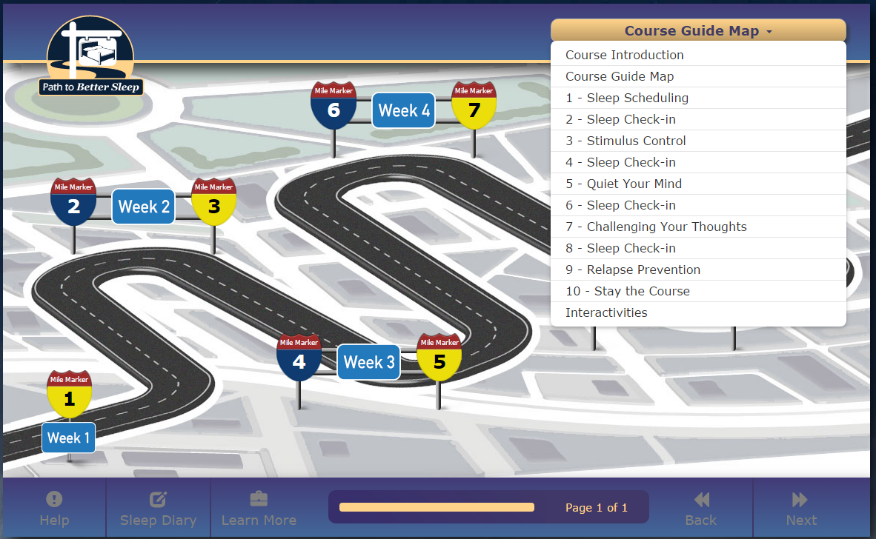
So, as you can see here, the meat of the course itself is split into Chapters or Mile Markers. The odd-numbered ones (the yellow road signs) are where a user of the course learns something new – Sleep Scheduling, Stimulus Control, Quiet Your Mind, Challenging Your Thoughts, etc. The even-numbered mile markers are check-ins. Here, users answer questions and input information about their own experiences with the previous chapter’s content. These check-ins are meant to be done weekly after tracking your sleep all week.

1. What are your general impressions of this “Course Guide Map”? Please take a moment to enter a few words into the chat, and then we’ll discuss your thoughts. These can be about layout, content, or clarity. (Elizabeth to call on a few people to elaborate.)

# PART 2: Feedback on Mile Marker #1: sleep scheduling (20 minutes)

Thank you for that feedback. Now, we’re going to look at one chapter of the therapeutic part of the course that is meant to be used in conjunction with the sleep tracking options we looked at last month. We’ll start at Mile Marker 1: Sleep Scheduling. We’re going to watch a very brief intro video, so please be sure your computer’s speakers are on and you have the volume up.


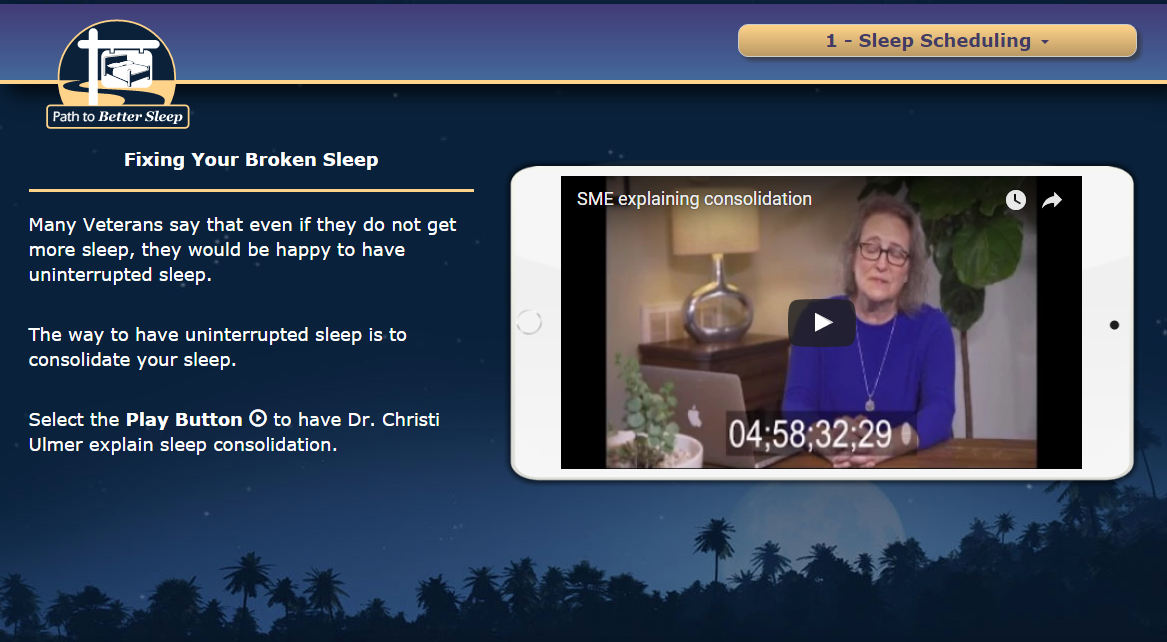
<http://www.sleepbettercourse.com/to2/index.html#02_01_005> - Sleep Scheduling. PLAY VIDEO

So we’ve just heard Dr. Ulmer explain some of the concepts that we saw in the Sleep Diary last month: “Sleep efficiency” being the biggest one. We’re going to move forward [NEXT PAGE] and go through some of the exercises that demonstrate how to calculate sleep efficiency and adjust your sleep schedule.

1.
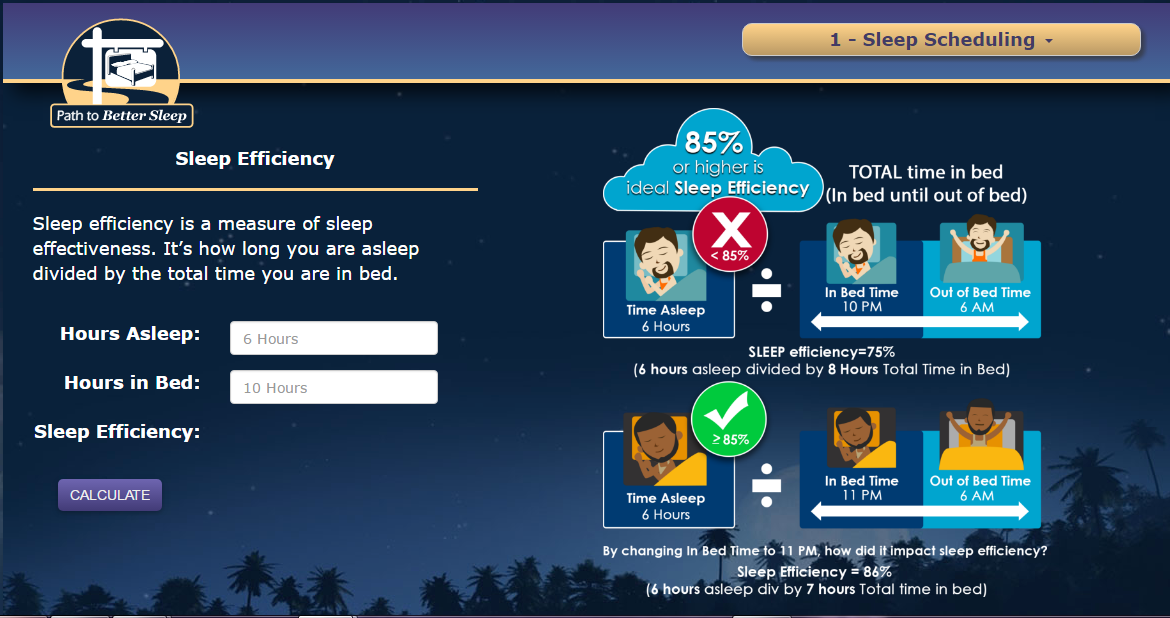
So I’d like you all to take a minute to look at this page on Sleep Efficiency. Have a read-through and please type into the chat box what you think “Sleep Efficiency” means, in your own words. Remember, we’re not testing YOU, we’re testing the explanations found in the course to make sure they are clear. (Elizabeth to call on a few people to elaborate.)


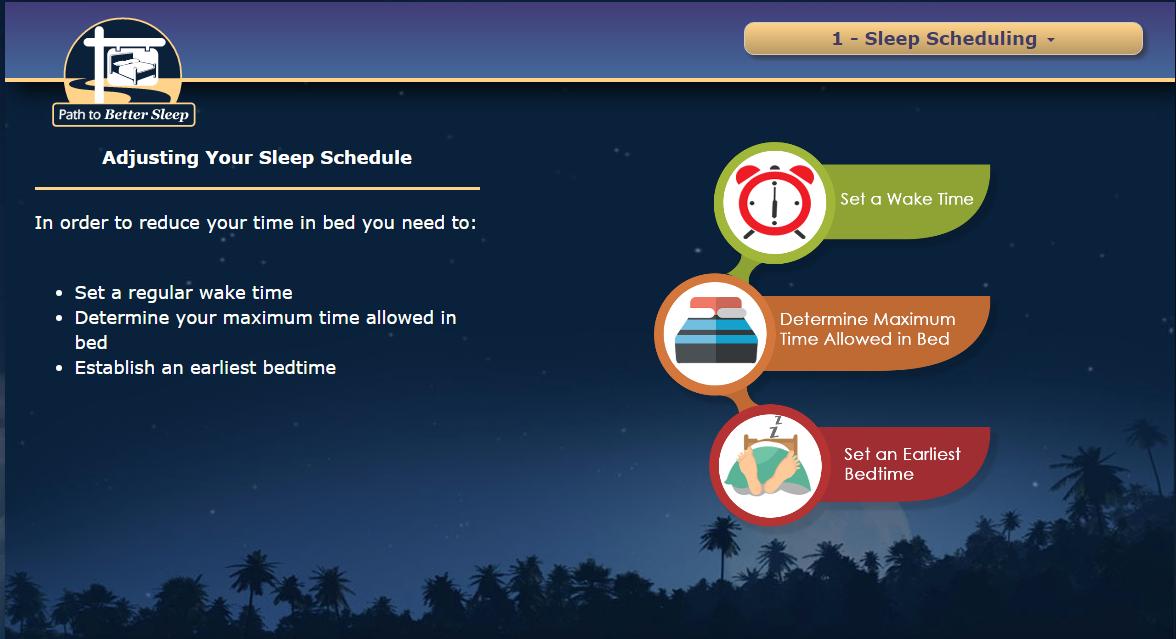
SKIP PAGE 4 – GO TO PAGE 5 – “Adjusting Your Sleep Schedule”

Thank you for that. Moving on to the next page: Adjusting your sleep schedule. Take a moment again to have a look and I’m just going to call on one or two people to get your initial impressions of the page, or feel free to chime in if you have anything to add.

(Elizabeth to call on 1 to 2 people for their impressions of the look/feel & organization)


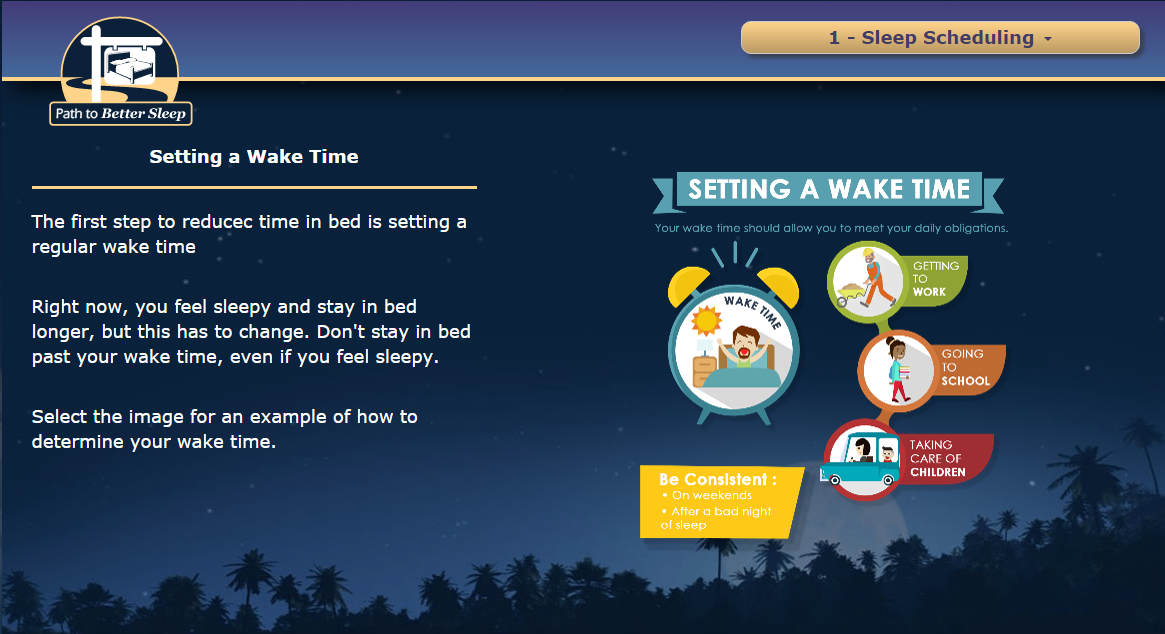
And now we’ll go to the “Setting a Wake Time” page. INTERACTIVITY DOESN’T WORK? SAYS TO SELECT THE IMAGE FOR AN EXAMPLE, BUT NOTHING HAPPENS. Not crucial…

1. I’d like you to please focus on the image on the right. What do you think of the clarity and the presentation of the information? How easy is it to read and understand?
   1. What are the main considerations in setting a wake time?

Thank you for that. I now want to take us to the practice scenarios. I know that not everyone here has struggled with insomnia or sleep disorders before. So I’d just like you to keep it front of your mind that this course is geared towards people who have trouble falling or staying asleep for weeks and months at a time. With that, let’s go through an exercise to help a couple of people with sleep issues set their wake times.


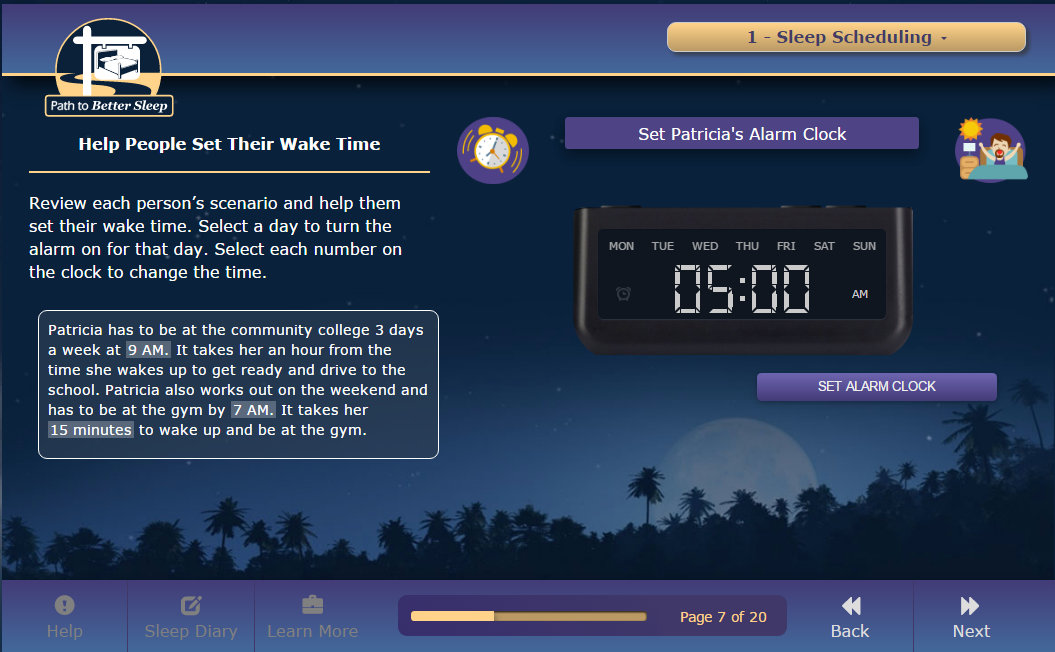
Read the text in the box (Patricia’s scenario).

1. Now please type into the chat box the days and times that we should choose for Patricia’s wake time. (Elizabeth to choose someone’s answer and fill out the clock accordingly. Recommend choosing a wrong or incomplete answer so that they can see what happens. Then choose the right answer: 6:45 am, 7 DAYS A WEEK.
   1. Discuss any areas that may have caused confusion: Consistent wake time every single day?
   2. Clarify if they would want the info from the previous screen reiterated on the alarm clock page or just presented more prominently on previous page?

Thanks so much for that feedback. There are a couple more example scenarios, but we’re going to move on to the next screens: “Filling in the Middle Pieces” and “Reducing Time”


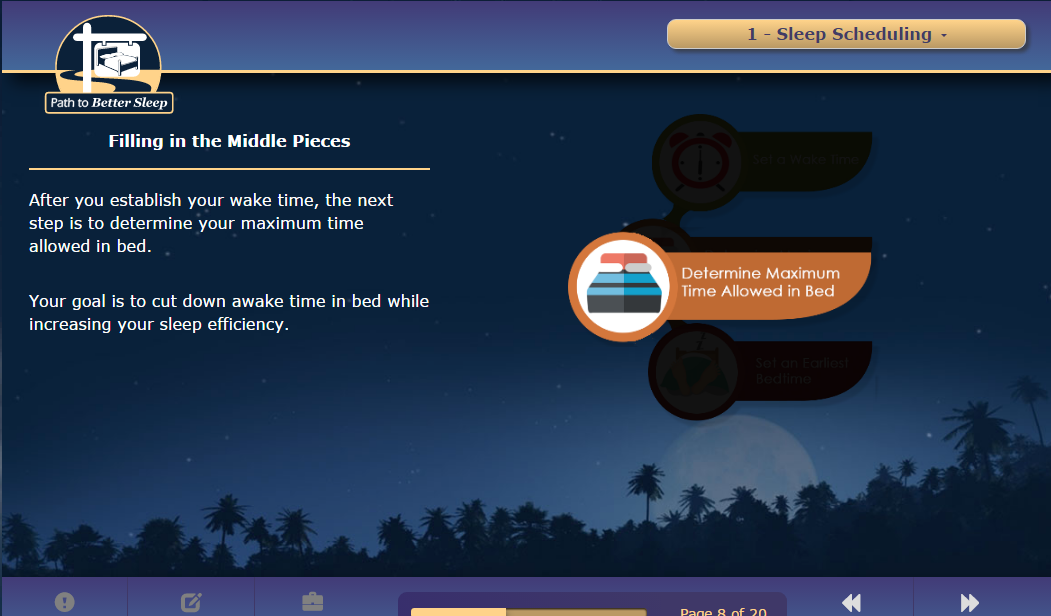
So this page discusses determining your “Maximum time allowed in bed”. This is something we need to figure out, in addition to the wake time.

1. What do you think is meant by “Maximum Time Allowed in Bed?”

And that then brings us to the next screen that will tie the sleep course together with the Sleep Diary we looked at last month. (Also, for those of you who mentioned sleep tracking devices like the FitBit, this part of the course will allow you to tie in sleep data from your FitBit with this Sleep Course).


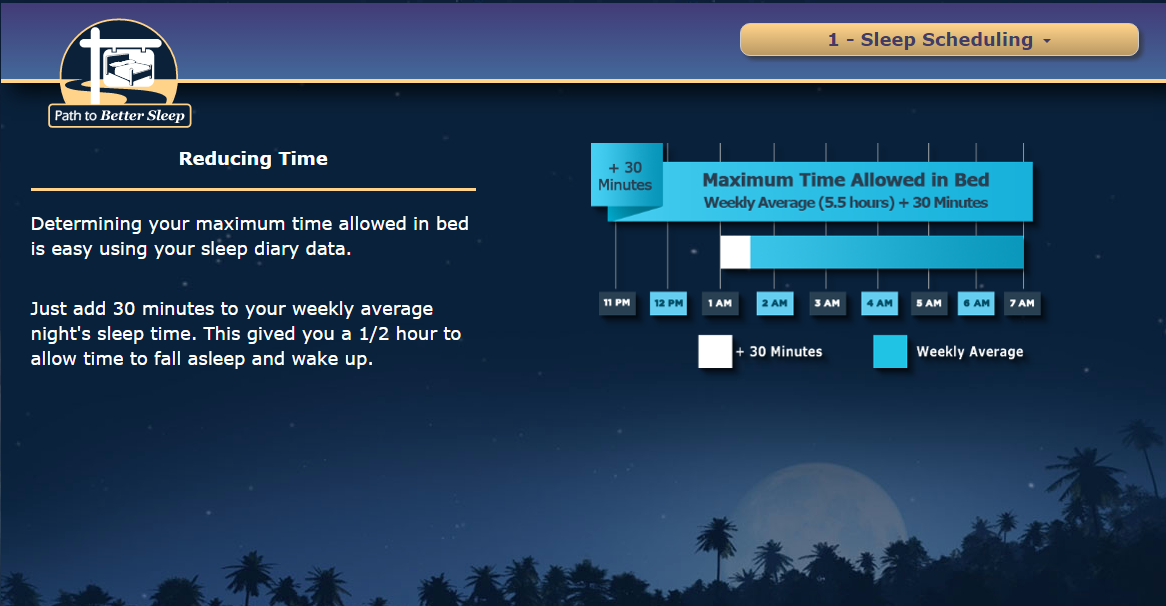


1. So, I’d like you to take a minute to really read this page. Then please let me know, in the chat box, what you’ve understood from these instructions. (Elizabeth to call on a few people to elaborate)
   1. When looking at this page, do you find the text on the left or the image on the right to be more useful or clear? Why?

Thank you all so much for that. This chapter goes into further details on scheduling your sleep and using your Sleep Diary data to see where you may be having issues so that you can improve your sleep efficiency. Obviously we can’t keep you all evening, so we won’t be going through the entire chapter. But having now seen both the Sleep Diary last month and part of the first chapter of the course this month as well as the course chapters laid-out on the Guide Map, what are your general impressions or feedback?

# Closing (2 minutes)

And now, as we conclude, is there anything final that anyone would like to add?

I’d just like to thank all of you for taking the time to share your thoughts with us over the past few months. Your feedback and input will be very helpful as we work to improve VA’s online self-help resources for Veterans.
